# Genomics-Enabled Novel Insight Into the Pathovar-Specific Population Structure of the Bacterial Leaf Streak Pathogen *Xanthomonas translucens* in Small Grain Cereals

**DOI:** 10.3389/fmicb.2021.674952

**Published:** 2021-05-28

**Authors:** Syed Mashab Ali Shah, Moein Khojasteh, Qi Wang, S. Mohsen Taghavi, Zhengyin Xu, Pejman Khodaygan, Lifang Zou, Sedighe Mohammadikhah, Gongyou Chen, Ebrahim Osdaghi

**Affiliations:** ^1^School of Agriculture and Biology/State Key Laboratory of Microbial Metabolism, Shanghai Jiao Tong University, Shanghai, China; ^2^Department of Plant Protection, School of Agriculture, Shiraz University, Shiraz, Iran; ^3^Department of Plant Protection, University of Tehran, Karaj, Iran; ^4^Department of Plant Protection, Faculty of Agriculture, Vali-e-Asr University of Rafsanjan, Rafsanjan, Iran

**Keywords:** bacterial leaf streak, Xanthomonas translucens, nanopore sequencing, genome structure, small grain cereals

## Abstract

The Gram-negative bacterium *Xanthomonas translucens* infects a wide range of gramineous plants with a notable impact on small grain cereals. However, genomics-informed intra-species population structure and virulence repertories of the pathogen have rarely been investigated. In this study, the complete genome sequences of seven *X. translucens* strains representing an entire set of genetic diversity of two pathovars *X. translucens* pv. *undulosa* and *X. translucens* pv. *translucens* is provided and compared with those of seven publicly available complete genomes of the pathogen. Organization of the 25 type III secretion system genes in all the 14 *X. translucens* strains was exactly the same, while TAL effector genes localized singly or in clusters across four loci in *X. translucens* pv. *translucens* and five to six loci in *X. translucens* pv. *undulosa*. Beside two previously unreported endogenous plasmids in *X. translucens* pv. *undulosa*, and variations in repeat variable diresidue (RVD) of the 14 strains, *tal1a* of *X. translucens* pv. *translucens* strain XtKm8 encode the new RVDs HE and YI which have not previously been reported in xanthomonads. Further, a number of truncated *tal* genes were predicted among the 14 genomes lacking conserved *Bam*HI site at N-terminus and *Sph*I site at C-terminus. Our data have doubled the number of complete genomes of *X. translucens* clarifying the population structure and genomics of the pathogen to pave the way in the small grain cereals industry for disease resistance breeding in the 21st century’s agriculture.

## Introduction

The Gram-negative phytopathogenic members of the genus *Xanthomonas* cause devastating diseases on hundreds of agricultural crops i.e., the members of *Poaceae* family, e.g., barley, maize, oat, rice, rye, sugarcane, triticale, and wheat (Jacques et al., 2016; Sapkota et al., 2020). Diseases caused by xanthomonads on gramineous crops comprise leaf streak, black chaff, blight, or wilt symptoms (Denancé et al., 2016). Bacterial leaf streak of small grain cereals caused by different pathovars of *X. translucens* is one of the economically important diseases of wheat and barley worldwide. The disease occurs in many countries across the globe with a particular importance in regions characterized by high precipitations. *Xanthomonas translucens* pv. *undulosa*, *X. translucens* pv. *translucens* and *X. translucens* pv. *cerealis* are the only member of nine pathovars within the species that are reported to have wide geographic distribution causing economic yield losses on wheat and barley (Sapkota et al., 2020). Hence, these pathovars are included in the A2 (high risk) list of quarantine pathogens by the European and Mediterranean Plant Protection Organization, and are under strict quarantine control and zero tolerance in several countries (EPPO, 1998; Sapkota et al., 2020). All the three pathovars possess broad host range where *X. translucens* pv. *cerealis* infects rye, oat, bromus and wheat; *X. translucens* pv. *translucens* infects barley, oat, rye and harding grass; and *X. translucens* pv. *undulosa* infects triticale, oat, rye, bromus, barley and wheat (Khojasteh et al., 2019). The remaining seven pathovars of the species are associated with different grasses having narrow host range with lower economic impact (Sapkota et al., 2020).

The *X. translucens* pathogens are seed-borne and can impact grain quantity and quality by reducing number of kernels per spike, and grain weight (Shane et al., 1987). Yield losses due to the bacterial leaf streak mainly depend on resistance/susceptibility of cultivars grown, environmental conditions and availability of primary inoculum (CABI, 2020; Sapkota et al., 2020). Despite the pivotal economic importance of the bacterial leaf streak in cereals industry, different pathovars of the pathogen have rarely been subjected to phylogenomics and comparative genomics in order to clarify molecular characteristics and virulence repertories of each of the above mentioned pathovars. Similar to the bacterial spot of solanaceous vegetables caused by four genetically distinct xanthomonads (Potnis et al., 2015; Osdaghi et al., 2016, 2017) determination of the exact yield lose corresponding to each of the small grains-pathogenic xanthomonads on wheat and barley is practically neither possible nor reliable. Unless otherwise exactly determined, the economic lose attributed to the bacterial leaf streak disease is usually considered as a whole. Recently, Khojasteh et al. (2019) have investigated the phylogenetic relationships and phylogeography of all the available *X. translucens* strains and proposed the Middle Eastern countries, i.e., Fertile Crescent as the center of diversity of the pathogen.

Plant pathogenic xanthomonads translocate a cocktail of different effector proteins into host plant cells referred to as type-III effectors (T3Es) using the type three secretion system (T3SS). The T3Es are further categorized into TALEs (transcription activator-like effectors) and non-TALEs which also known as Xops (*Xanthomonas* outer proteins). It has been shown that the bacterial leaf streak pathogen isolated from barley and wheat crops possess high genetic diversity in terms of TALEs (Khojasteh et al., 2020). During the past decade, high throughput complete genome sequencing technologies have provided substantial progresses in the understanding of molecular mechanisms underlying plant colonization, pathogenicity and survival of the bacterial leaf streak pathogens (Sapkota et al., 2020). Genome-informed investigation of *X. translucens* species complex provides valuable information on the virulence repertories, pathogenicity mechanisms, and host adaptation of the bacterial leaf streak pathogens (Peng et al., 2016). Until 2021, complete genome resources of seven *X. translucens* strains, i.e., *X. translucens* pv. *cerealis* strains CFBP 2541 and NXtc01 (Pesce et al., 2015; Shah et al., 2019), *X. translucens* pv. *translucens* strain DSM 18974^T^ (Jaenicke et al., 2016), and *X. translucens* pv. *undulosa* strains Xtu 4699, ICMP 11055, P3 and LW16 (Peng et al., 2016, 2019; Falahi Charkhabi et al., 2017) were publicly available. Using these genome resources, it has been shown that TALEs have pivotal contribution to the virulence and adaptation of the bacterial leaf streak pathogens facilitating host plant colonization, fitness and proliferation within the host plant tissues. So far, three TALEs, i.e., ICMP 11055 Tal2 and Tal4b, and NXtc01_Tal1 have been reported to have effective contribution to the virulence of the bacterial leaf streak pathogens (Falahi Charkhabi et al., 2017; Shah et al., 2019). Recently, a TALE named Xtu 4699_Tal8 has functionally been characterized, inducing expression of wheat gene TaNCED located on short arm of chromosome 5B to promote disease susceptibility (Peng et al., 2019). Despite the abundance of data on geographic distribution and genetic diversity of the bacterial leaf streak agents (Curland et al., 2018; Khojasteh et al., 2020), complete genome sequence-based population structure and genomic repertories of the pathogens mostly remain uninvestigated.

In this study, in order to provide a comprehensive insight into the population structure, genomic content and pathogenicity determinants of *X. translucens*, we have selected seven *X. translucens*, i.e., four X. *translucens* pv. *undulosa* and three *X. translucens* pv. *translucens* strains among a collection of 57 strains isolated in Iran during the past couple of decades (Khojasteh et al., 2019). The representative strains were selected based on their host range and pathovar status, multilocus sequence analysis and typing (MLSA/MLST) scheme as well as the Southern blot-based TALE diversity as described previously (Khojasteh et al., 2020). The strains were sequenced using Oxford Nanopore PromethION long-read direct DNA sequencing platform. To evaluate the genomic variations among and between *X. translucens* pathovars we applied a comparative genomic workflow, taking into account the complete genome sequences of a set of seven reference strains. Complete genome sequencing revealed that the *X. translucens* pv. *undulosa* strains XtKm15 and XtLr8 each harbors two plasmids which have not previously been reported in any strain of this pathovar. We analyzed all the genomes for novel genes that might be important for pathogenicity, particularly TAL and non-TAL effectors with homologs in other *Xanthomonas* strains. These data provided an important insight into the *X. translucens*-gramineous crops pathosystem and pave the way for future development of resistant cultivars.

## Materials and Methods

### Bacterial Strains, Growth Conditions and Genomic DNA Extraction

The bacterial strains used in this study are listed in Table 1. A set of seven *X. translucens* strains, i.e., four *X. translucens pv. undulosa* strains: XtKm12, XtKm15, XtLr8, and XtFa1 and three *X. translucens* pv. *translucens* strains: XtKm8, XtKm9, and XtKm34 were selected from a collection of 57 strains isolated from wheat and barley in Iran during 2008 to 2017 (Khojasteh et al., 2019). The criteria used for selection of the strains were their host range and pathovar status, MLSA-based genetic diversity and TALE repertories of the strains (Khojasteh et al., 2020). The bacterial strains were streaked onto nutrient agar (NA) medium or nutrient broth medium (NB: NA without agar) when required and incubated at 28°C. All the strains were stored at −80°C in nutrient broth (NB) medium amended with 50% sterile glycerol. The genomic DNA of the bacterial strains was extracted from a 24 h culture in NB medium using the Hipure bacterial DNA extraction kit (Magen, Guangzhou, Guangdong, China) as recommended by the manufacturer. The quality and quantity of the DNAs were spectrophotometrically evaluated and adjusted to 1500 ng/μL using the NanoDrop ND-100 (NanoDrop Technologies, Waltham, MA, United States) and then confirmed by 1.0% agarose gel electrophoresis.

**TABLE 1:** Genomic characteristics of *Xanthomonas translucens* strains investigated in this study. The first seven strains were sequenced in this study while the next seven reference strains were retrieved from the NCBI GenBank.

	**Strains sequenced in this study**							**Reference sequences obtained from the NCBI GenBank**						
	**XtFa1**	**XtLr8**	**XtKm12**	**XtKm15**	**XtKm8**	**XtKm9**	**XtKm34**	**NXtc01**	**ICMP 11055**	**CFBP 2541**	**DSM 18974**^**T**^	**LW16**	**P3**	**Xtu 4699**
Host	Wheat	Wheat	Wheat	Ryegrass	Barley	Barley	Barley	Wheat	Wheat	Bromegrass	Barley	Wheat	Wheat	Wheat
Region	Fars	Lorestan	Kerman	Kerman	Kerman	Kerman	Kerman	Xinjiang	Kerman	–	Minnesota	North Dakota	North Dakota	Kansas
Country	Iran	Iran	Iran	Iran	Iran	Iran	Iran	China	Iran	United States	United States	United States	United States	United States
Year	2016	2016	2015	2015	2014	2015	2015	2016	1983	1941	1933	2009	2009	1999
Genome length (bp)	4,605,208	4,563,212	4,581,137	4,560,646	4,792,950	4,689,955	4,680,513	4,622,298	4,761,583	4,518,140	4,715,357	4,746,074	4,618,583	4,561,137
G + C content (%)	68.06	68.04	68.01	68.04	67.79	67.87	67.79	67.23	67.8	67.34	67.7	67.8	68.1	68.1
Protein-coding genes	3,699	3,731	3,654	3,728	3,873	3,763	3,768	3,733	3,835	3609	3804	3752	3723	3636
RNA genes	63	63	63	63	63	64	63	64	63	60	64	64	63	63
Pseudo-genes	218	211	205	211	208	200	185	256	224	294	190	234	215	205
CRISPR arrays	1	1	1	1	1	1	1	1	1	3	1	1	1	ND
TAL effector genes	7	8	7	8	8	5	7	2	7	2	8	8	8	8
Non-TAL T3E genes	30	29	31	29	34	36	33	35	27	31	36	29	30	27
Genome coverage (×)	478	490	313	475	445	484	385	80	118	1926	NA	90	90	60
Acc. no.	CP063996	CP063993	CP064000	CP063997	CP064004	CP064003	CP064001	CP038228	CP009750	NZ_CM003052	LT604072	CP043540	CP043500	CP008714
														

### Complete Genome Sequencing, Assembly, and Annotation

Genomic DNA of all *X. translucens* strains were sequenced using long-read Nanopore sequencing technology plus NovaSeq 6000 sequencing (OE Biotech Co. Ltd., Shanghai, China). Sequencing libraries were prepared and added to PromethION flow cells and transferred into the Oxford Nanopore sequencer for real-time single molecule sequencing. The NovaSeq 6000 short reads were also produced for assembly and polished by using Racon v1.4.3 (Vaser et al., 2017) and Pilon v1.22. For demultiplexing ONT-guppy v4.0.11 was used for promethion sequencing. After demultiplexing, the obtained reads were assembled using Flye v2.5 (Kolmogorov et al., 2019) and Canu v1.7 (Koren et al., 2017) command-line services via default parameters. Nanopore data was first mapped to the assembled genomes with Minimap2-2.9 (Li, 2016) and then corrected three times with Racon v1.4.3. The Nanopore raw data of XtKm9, XtKm34, XtFa1, XtKm12, XtKm15 and XtLr8 were assembled using Flye with default parameters, while Canu (with default parameters) was used for XtKm8. Then all the assembled genomes were corrected three times via Minimap2+racon. Finally, all the genomes were polished three times with Pilon (v1.22) (Walker et al., 2014) using the high-quality short reads (generated by Trimmomatic-0.36; Bolger et al., 2014). Subsequently, genome annotation was performed using the GeneMarkS^+^ v4.6 suite implemented in the NCBI Prokaryotic Genome Annotation Pipeline with default settings (Borodovsky and Lomsadze, 2014). The assembled genomes were masked via RepeatMasker v4.0.7 (Tarailo-Graovac and Chen, 2009). For functional classification, the putative genes were annotated against five databases, i.e., KEGG, NR, COG, Swiss-Prot and GO with default parameters as described previously (Chen et al., 2020). Further, tRNA and rRNA genes were predicted using tRNAscan-SE v1.3.1 (Lowe and Eddy, 1997) and rRNAmmer v1.2 (Lagesen et al., 2007), respectively, while sRNAs were predicted using BLAST against the Rfam database (Griffiths-Jones et al., 2003). CRISPR sequences were predicted using PILER-CR v1.06 (Buchfink et al., 2015) and CRT1.2-CLI (Eddy, 2009). Prophages were also predicted using PhiSpy v2.3 (Akhter et al., 2012). The circular genome maps were generated using Circos to show annotation information.

### Phylogenomics, Comparative Genomics, and Pan-Genome Analysis

In order to determine the precise phylogenetic position of the strains sequenced in this study, all the publicly available genome sequences assigned as *X. translucens* (up to November 2020) were retrieved from the NCBI GenBank database and included in the phylogenetic analyses. Average nucleotide identity (ANI) was calculated among all *X. translucens* genome sequences using both one-vs.-one and all-vs.-all strategies via different algorithms, i.e., JSpeciesWS, ANI calculator, and OrthoANIu as detailed previously (Osdaghi et al., 2020). ANI calculator estimates all-vs.-all distances in a collection of genomes and builds a similarity clustering (Rodriguez-R and Konstantinidis, 2016). The OrthoANIu algorithm is an improved iteration of the original OrthoANI algorithm which uses USEARCH instead of BLAST (Yoon et al., 2017). JSpecies Web Server (JSpeciesWS) online service measures the ANI based on BLAST + (ANIb) and MUMmer (ANIm), as well as correlation indexes of tetra-nucleotide signatures (Richter et al., 2016). ANI-based Neighbor-Joining distance clustering plot was constructed using the ANI calculator online service for all the *X. translucens* strains. Further, to determine the gene pool of seven Iranian *X. translucens* strains and to compare these strains to the publicly available complete genomes of reference strains, the complete genome data of seven *X. translucens* strains, i.e., *X. translucens* pv. *cerealis* strains CFBP 2541 and NXtc01 (Pesce et al., 2015; Shah et al., 2019), *X. translucens* pv. *translucens* strain DSM 18974^T^ (Jaenicke et al., 2016), and *X. translucens* pv. *undulosa* strains Xtu 4699, ICMP 11055, P3 and LW16 (Peng et al., 2016, 2019; Falahi Charkhabi et al., 2017) were retrieved from the NCBI GenBank database and included in all the subsequent analyses. We carried out a pan-/core-genome analysis and functional assignment to the COGs categories by Roary 3.8.0 using the procedure described by Page et al. (2015). In brief, FASTA files of all the 14 *X. translucens* strains were transformed to GFF3 format using Prokka (Seemann, 2014) to create a nucleotide alignment using Roary 3.8.0 (with a 95% BLASTp percentage identity cut-off) to cluster the genes into core and accessory genomes. Phandango (Hadfield et al., 2018) and R packages including seqinR (Charif and Lobry, 2007) as well as tidyverse (Wickham et al., 2019) were applied to visualize the resulted output graphs. IslandViewer 4 was used for the identification and visualization of genomic islands (Bertelli et al., 2017) while pairwise genome collinearity alignment and visualization of the seven strains sequenced in this study was performed using BRIG 0.95 (Alikhan et al., 2011). The complete genome sequences of the strains DSM 18974^T^ and Xtu 4699 were used as reference genomes for *X. translucens* pv. *translucens* and *X. translucens* pv. *undulosa* strains, respectively. Further, Mauve software was used to illustrate locally collinear blocks among the genomes obtained in this study and those of the reference strains in the two pathovars (Darling et al., 2010). Genome-wide comparisons and visualization of orthologous clusters were performed using the online service OrthoVenn (Wang et al., 2015).

### Type III Secretion System Repertory

Database searches were performed for the prediction of genes encoding different secretion systems including type III secretion system (T3SS) and type III effectors (T3Es) in *X. translucens* genomes using KEGG orthologies (KO) by implementing KofamKOALA (Aramaki et al., 2020) and BLASTn/BLASTp as described previously (Peng et al., 2016; Pesce et al., 2017). In brief, the sequences of 63 T3Es were retrieved from http://xanthomonas.org/ and two T3Es, i.e., XopE4 and XopE5 were obtained from EuroXanth DokuWiki. A dataset including the information of all 65 T3Es are shown in the Supplementary Dataset 1. For further confirmation, all annotated effector sequences were searched through NCBI GenBank, https://www.uniprot.org/, http://xanthomonas.org/ and EuroXanth DokuWiki, and all the genome sequences were analyzed one-vs.-one using BLAST or PSI-BLAST. Amino acid sequences (BLASTp) were used in all the analyses while nucleotide sequences (BLASTn) were also implemented in the investigations for further confirmation. For BLASTP, *e*-value = 1e-5 with a 50% query coverage and 35% sequence similarity were considered as cut-off criteria as recommended previously (Pearson, 2013; Wichmann et al., 2013). Ortho MCL v. 2.0 was used to generate groups of orthologous proteins with default parameters.

### TALE Repertory of *X. translucens*

The genome sequences of 14 *X. translucens* strains were used for TALE prediction and TALE-based phylogenetic analysis. DisTAL v1.1 was used to align and phylogenetically classify TALEs based on their repeat arrangement (Pérez-Quintero et al., 2015). For the analysis of TALEs repeat variable diresidue (RVDs), we used AnnoTALE v1.2 that contained 516 *TALE* genes from 33 *Xanthomonas* strains (up to December 2020). First we analyzed all the *X. translucens* genomes and merged the TALEs RVDs output file into publicly available 516 TALEs RVDs. Then, the TALEs were grouped into different classes on the basis of RVDs that indicates their possible functional and evolutionary relationships (Grau et al., 2016; Erkes et al., 2017). TALE-CRR (central repeat region)-based tree was generated using DisTAL v1.1 with default parameters (Pérez-Quintero et al., 2015), while the resulting tree was visualized using FigTree v1.4.4 (Rambaut and Drummond, 2012). Furthermore, TALE repertory of the seven strains sequenced in study which have previously been investigated by Southern blotting of *Bam*HI-digested genomic DNAs (Khojasteh et al., 2020) was confirmed using complete genome sequence data.

### Data Availability

The dataset produced in this complete genome sequencing project is available at the NCBI GenBank/DDBJ/EMBL database under the accession numbers CP063993, CP063994, CP063995, CP063996, CP063997, CP063998, CP063999, CP064000, CP064001, CP064003, and CP064004 as detailed in Table 1.

## Results

### The Genome of *X. translucens*

Complete genome sequencing of *X. translucens* strains were performed using Oxford Nanopore PromethION platform. The assembled sequences of all seven strains consisted of a single circular chromosome. Interestingly, in two of the seven *X. translucens* strains sequenced in this study, i.e., XtKm15 and XtLr8 two plasmids were found. The plasmids of the strain XtKm15 were designated as XtKm15_P1 (41,956 bp) and XtKm15_P2 (45,639 bp), while the two plasmids in XtLr8 were designated as XtLr8_P1 (45,351 bp) and XtLr8_P2 (40,770 bp) as detailed in Table 1. General features of the *X. translucens* sequences obtained in this study as well as the seven reference complete genome sequences retrieved from the GenBank are comparatively presented in Table 1. Genome size in the strains sequenced in this study ranged from 4,560,646 bp in XtKm15 to 4,792,950 bp in XtKm8, while the GC% content of the strains was from 67.79% in XtKm8 and XtKm34 to 68.06% in XtFa1. The number of protein coding genes ranged between 3,654 in XtKm12 to 3,873 in XtKm8. Supplementary Figure 1 shows the circular diagram and genome features of the seven chromosomes and four plasmids resulted from this genome sequencing project. BLASTn-based investigation showed non-significant (<2% query coverage) similarities between the genome sequences of XtKm15 and XtLr8 and their accompanying plasmids (data not shown). However, the plasmid XtKm15_P1 had 92% query coverage and 99.5% sequence identity with XtLr8_P2 while XtKm15_P2 had 86% query coverage and 99.5% sequence identity with XtLr8_P1.

### Phylogeny of *X. translucens*

**FIGURE 1:**
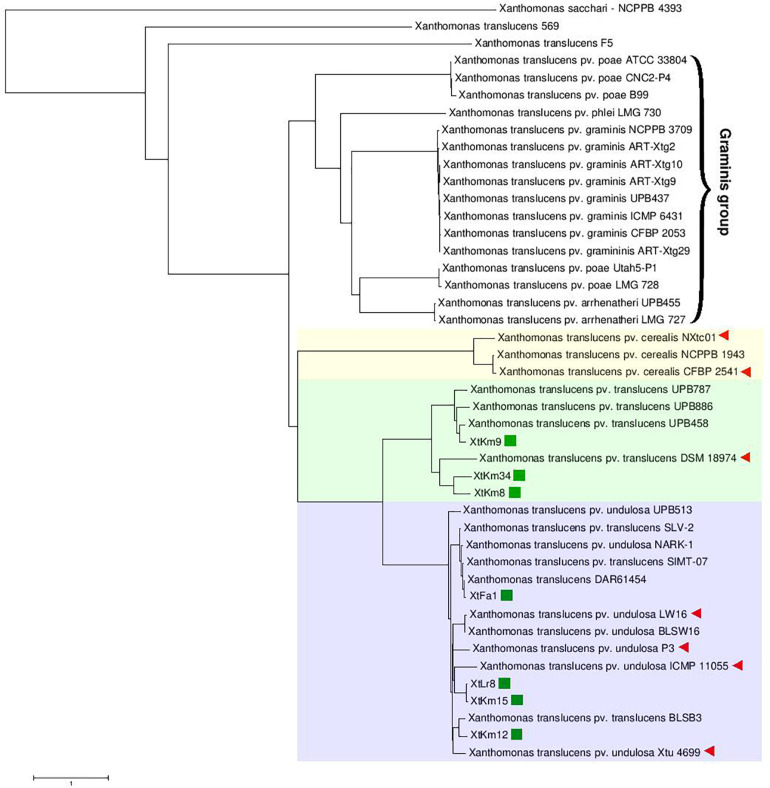
Average Nucleotide Identity (ANI)-based Neighbor-Joining distance clustering plot of all available *Xanthomonas translucens* strains constructed using the ANI calculator online service. Members of *X. translucens* were clustered in two phylogenetically distinct clades, i.e., graminis and translucens groups. Among the strains sequenced in this study, XtKm8, XtKm9, and XtKm34 clustered within the *X. translucens* pv. *translucens* clade while the strains XtFa1, XtLr8, XtKm12, and XtKm15 clustered in the *X. translucens* pv. *undulosa* clade. Three strains, i.e., SLV-2, SIMT-07, and BLSB3 that labeled as *X. translucens* pv. *translucens* in the NCBI GenBank database were clustered within the *X. translucens* pv. *undulosa* strains indicating a mislabeling in the identification of these strains. Based on the ANI values, the strains 569 and F5 could not be included within *X. translucens*, where the ANI values between these two strains and the other members of the species were consistently below 93%.

Neighbor-joining distance clustering plot constructed using the genome sequences of 43 publicly available *X. translucens* whole genome sequences via ANI Calculator online service with all-vs.-all strategy revealed high genetic diversity among *X. translucens* complex species. Members of the three pathovars, i.e., pv. cerealis, pv. translucens, and pv. undulosa which are commonly referred to as translucens group were clustered in a monophyletic clade including all the 14 completed genome sequences investigated in this study (Figure 1). ANI values among the members of each of the translucens and graminis groups were higher than those observed between the members of the two groups (Supplementary Figure 2). However, the two clades *X. translucens* pv. *undulosa* and *X. translucens* pv. *translucens* were phylogenetically closer to one another while *X. translucens* pv. *cerealis* strains were clustered in a distinct clade. The three *X. translucens* pv. *translucens* strains sequenced in this study, i.e., XtKm8, XtKm9, and XtKm34 were clustered in two subclades where XtKm8 and XtKm34 were close to the reference strain DSM 18974^T^ while XtKm9 was clustered with the other three strains of this pathovar. Surprisingly, three strains, i.e., SLV-2, SIMT-07, and BLSB3 that are labeled as *X. translucens* pv. *translucens* in the NCBI GenBank database were clustered within the *X. translucens* pv. *undulosa* strains indicating a mislabeling in the identification of these strains. The four *X. translucens* pv. *undulosa* strains sequenced in this study, i.e., XtFa1, XtLr8, XtKm12, and XtKm15 were scattered through different subclades indicating that these strains are a proper set of representatives within the pathovar which were selected on the basis of MLSA results. As for the graminis group of *X. translucens*, all the strains designated as *X. translucens* pv. *graminis* were clustered in a monophyletic clade as well as the *X. translucens* pv. *arrhenatheri* strains. However, the five strains designated as *X. translucens* pv. *poae* were divided into two phylogenetically distinct clades where the two strains Utah5-P1 and LMG 728 were placed close to the *X. translucens* pv. *arrhenatheri* clade while the three strains ATCC 33804, CNC2-P4, and B99 were clustered in a distinct clade apart from the other members of graminis group as shown in Figure 1. Furthermore, the strains 569 and F5 have not been included in either group of *X. translucens* where the ANI values between these two strains and the other members of *X. translucens* were consistently below 93%, suggesting that the strains 569 and F5 could not be considered as *X. translucens*. Tacking together, a formal and comprehensive taxonomic study is warranted to address the two last taxonomic issues within *X. translucens* members and further refine the classification of this complex species.

### Comparative Genomics

**FIGURE 2:**
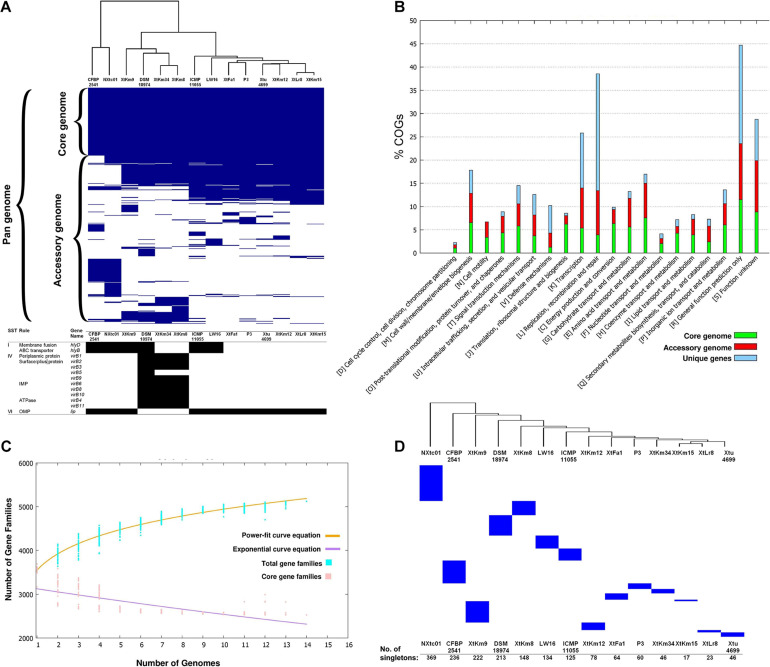
Core- vs. pan-genome analyses of 14 *Xanthomonas translucens* strains using different criteria. **(A)** Ratio and size plot of core- and pan-genome generated using Roary 3.8.0 and visualized with Phandango. Each column corresponds to one strain while each row represents an orthologous gene family. The black and white table at the bottom of core- vs. pan-genome heat map created using KofamKOALA where black and white squares indicate presence and absence of a certain gene, respectively. **(B)** COG distribution of core, accessory and unique genes in the 14 *X. translucens* strains obtained using BPGA v. 1.3. **(C)** Total number of distinct gene families referring to the pan-genome of *X. translucens* strains illustrated by BPGA v. 1.3. **(D)** The number of singletons in each of the 14 *X. translucens* strains obtained using Roary 3.8.0.

The core-genome of 14 *X. translucens* strains investigated in this study consisted of 2,175 genes appeared in >99% of the strains with >95% sequence similarity, while no soft core genes (presented in 95% to 99% of the strains) was detected. The number of shell genes presenting in 15% to 95% of the 14 strains was 2,384, while the number of cloud genes that found in 0% to 15% of the strains was 3,022. The pan-genome (total genes) of the 14 *X. translucens* strains was determined as 7,581 (Figure 2A). Distribution of the clusters of orthologous groups (COG) affiliated to biological functions is shown in Figure 2B. The highest proportion of unique genes in a certain COG was found in the orthologous groups assigned to replication, recombination and repair (L), and general function (R) making 65% and 48% of the total genes, respectively; followed by transcription (K) cluster. The least number of unique genes was found in translation, ribosomal structure and biogenesis (J) and energy production and conversion (C) orthologous groups, while no unique gene was found in the cell motility (N) COG. The highest number of core genes was predicted in the general function cluster (R) while the COGs assigned to defense mechanisms (V) showed the least number of core genes. The number of accessory genes with unknown function (S), cell wall/membrane/envelope biogenesis (M) and intracellular trafficking, secretion, and vesicular transport (U) were proportionally similar to those of the core genes and unique genes in their respective COG as shown in Figure 2B. Translation, ribosomal structure and biogenesis (J) COG along with the coenzyme transport and metabolism (H) had the least number of accessory genes.

The core- vs. pan-genome plot generated with BPGA 1.3v software revealed an open pan-genome for the 14 *X. translucens* strains investigated in this study. Figure 2C represent the power-fit curve resulted from the equation [*f*(*x*) = *a*.*x*^*b*], where the exponent *b* > 0 indicates that the genome is open (Bosi et al., 2015). Therefore, the number of dispensable or accessory genes increases with the increase in the number of genomes indicating that the pan-genome of *X. translucens* has not yet been closed (Figure 2C). The “*b* = 0.143” indicates an open pan-genome for the 14 *X. translucens* but may be closed soon. In the other word, the unique gene pool should be increased by addition of newly sequenced *X. translucens* genomes. The pan-genome expansion analysis is biased and limited by the number of strains and pathovars used in the analysis. The number of singletons (genes unique to a single strain) in each of the 14 *X. translucens* strains is presented in Figure 2D. Considering the entire dataset, the highest number of singletons was found in the strain NXtc01 (369 singletons) followed by CFBP 2541 (236 singletons) both belonging to *X. translucens* pv. *cerealis*. As for the strains sequenced in this study, the strain XtKm9 had the highest number of singletons (222) followed by XtKm8 (148 singletons) both belonging to *X. translucens* pv. *translucens*. The least number of singletons was found in XtKm15 (17 singletons) followed by XtLr8 (23 singletons) both belonging to *X. translucens* pv. *undulosa*, and the *X. translucens* pv. *translucens* strain XtKm34 (46 singletons).

**FIGURE 3:**
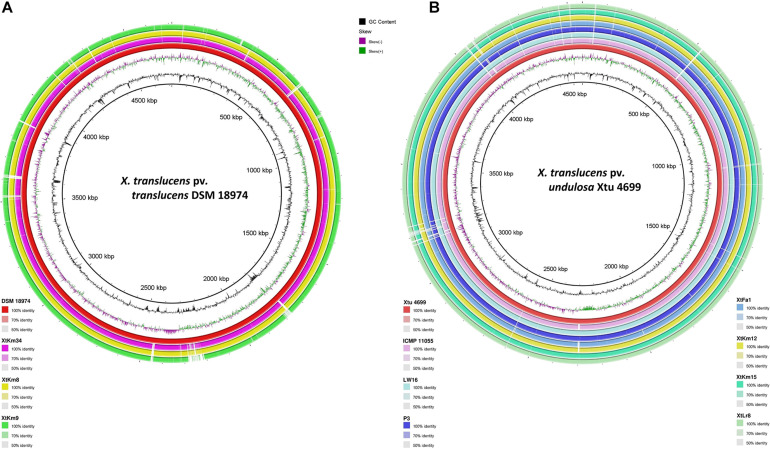
Pairwise genome collinearity alignment of the seven *Xanthomonas translucens* strains sequenced in this study performed using BRIG 0.95. The genomes of DSM 18974^T^ **(A)** and Xtu 4699 **(B)** were used as reference genomes for *X. translucens* pv. *translucens* and *X. translucens* pv. *undulosa* strains, respectively.

**FIGURE 4:**
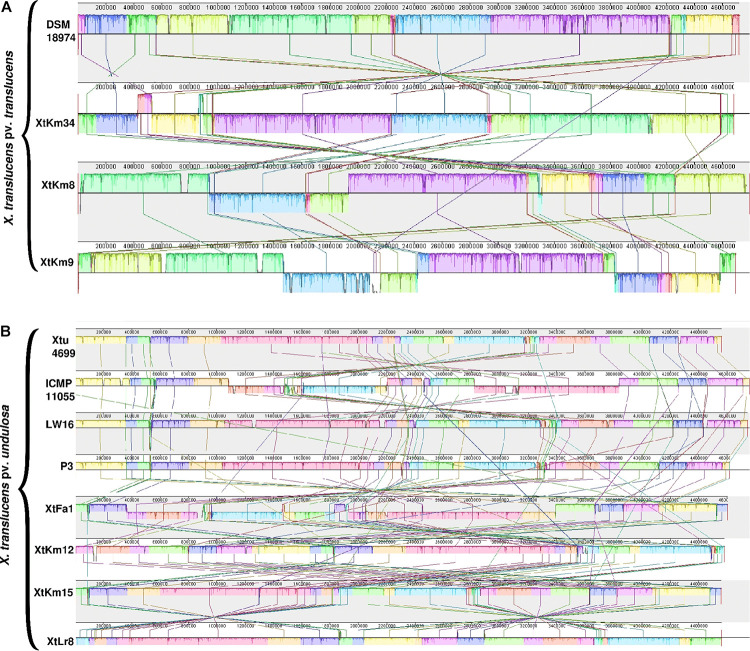
Pairwise alignment among the chromosomal DNA of *Xanthomonas translucens* strains sequenced in this study and the strains DSM 18974^T^ and Xtu 4699 as reference genomes for **(A)** *X. translucens* pv. *translucens* and **(B)** *X. translucens* pv. *undulosa* strains, respectively using MAUVE software. Colors show conserved and highly related genomic regions (locally collinear blocks). Blocks shifted below the center line indicate segments that align in the reverse orientation as inversions relative to the respective reference strain. Each contiguously colored region is a locally collinear block, which is a region without rearrangement of homologous backbone sequence. Lines between two genomes trace each orthologous locally collinear block.

Distribution of genomic islands - part of a genome that has evidence of having horizontal origins - in the sequences of seven strains obtained in this study and their respective reference genomes is shown in Supplementary Figure 3. The largest genomic islands were detected in the strain ICMP 11055 while most of the strains possessed a unique pattern of island distribution in their genome. In order to provide a comparative scheme for the genomes obtained in this study with their respective reference genomes, BRIG 0.95 was used to whole genome-based comparative genomics using DSM 18974^T^ and Xtu 4699 as reference genomes for *X. translucens* pv. *translucens* and *X. translucens* pv. *undulosa*, respectively (Figures 3A,B). Three *X. translucens* pv. *translucens* strains sequenced in this study lacked the genomics islands detected in DSM 18974^T^ (Figure 3A). For instance, a large island between the positions of 2,200 kbp and 2,500 kbp in DSM 18974^T^ was lacking in all the three *X. translucens* pv. *translucens* strains sequenced in this study. As for the *X. translucens* pv. *undulosa* strains, a large fraction of a genomic island in the position of 3,100–3,300 kbp was lacking in the four strains sequenced in this study. The islands between 3,900 kbp and 4,100 kbp in the genome of Xtu 4699 were also lacking in the query strains (Figure 3B). One-vs.-all collinearity test was performed among the genomes obtained in this study and their respective reference genomes using Mauve, where the organization of locally collinear blocks (LCBs) determined genome rearrangements and segmentation (Figures 4A,B). In the *X. translucens* pv. *translucens* genomes, the order of LCBs in the strain XtKm34 was almost entirely in congruence with the LCBs in the reference genome DSM 18974^T^ except for a reversion in a 100 kbp (nucleotides 400–500 kbp) fragment in XtKm34. The strains XtKm8 and XtKm9 showed more variations in their LCB arrangement where almost 40% of the XtKm9 genome experienced a reversion as shown in Figure 4A. As for *X. translucens* pv. *undulosa*, the strains XtKm12, XtKm15, and XtLr8 had the LCB arrangement similar to the reference strain Xtu 4699. However, the genome of XtFa1 had multiple translocations, inversions and rearrangements (Figure 4B). Each LCB is a homologous region of sequence shared by the reference genome and the genomes under study with no rearrangements of homologous sequence. Hence, from the functional point of view it would be more probable for a LCB to have similar biological feature in their corresponding genomes.

**FIGURE 5:**
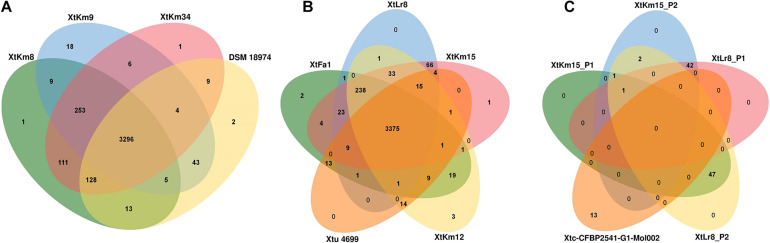
Venn diagrams constructed using the OrthoVenn online service showing the shared gene families (orthologous clusters) among different sets of strains. The numbers indicate the number of shared gene families among certain set of genomes. **(A)** *X. translucens* pv. *translucens* strains, **(B)** *X. translucens* pv. *undulosa* strains, and **(C)** plasmids XtKm15_P1 and XtKm15_P2 associated with the strain XtKm15 as well as XtLr8_P1 and XtLr8_P2 associated with the strain XtKm8. The genomes of DSM 18974^T^ and Xtu 4699 were used as reference genomes for *X. translucens* pv. *translucens* and *X. translucens* pv. *undulosa* strains, respectively while the plasmid Xtc-CFBP 2541-G1-Mol002 associated with *X. translucens* pv. *cerealis* strain CFBP 2541 was used as reference for the four plasmids.

Orthologous gene clusters were determined using OrthoVenn online service through four-vs.-four and five-vs.-five designations of the strains as shown in Figure 5. Three *X. translucens* pv. *translucens* strains sequenced in this study shared 3,296 proteins with the reference genome DSM 18974^T^, while the strains XtKm8, XtKm9, and XtKm34 each had one, eighteen, and one unique proteins in their genomes (Figure 5A). As for the *X. translucens* pv. *undulosa*, the four strains sequenced in this study showed 3,375 shared proteins with the reference strain Xtu 4699. The strains XtFa1, XtLr8, XtKm12 and XtKm15 had two, zero, three and one unique proteins, respectively (Figure 5B). The protein contents of the four plasmids identified in the strains XtLr8 (i.e., XtLr8_P1 and XtLr8_P2) and XtKm15 (i.e., XtKm15_P1 and XtKm15_P2) were evaluated against the previously reported plasmid Xtc-CFBP 2541-G1-Mol002 in *X. translucens* pv. *cerealis* CFBP 2541. Surprisingly, only one protein sequence was found to be shared among the four plasmids identified in this study with no unique protein in each of the four plasmids. The two plasmids XtLr8_P1 and XtKm15_P2 had 42 shared proteins hypothecating their similar origin. The plasmids XtLr8_P2 and XtKm15_P1 had also 47 shared proteins. The reference plasmid Xtc-CFBP 2541-G1-Mol002 showed 13 unique proteins suggesting its genomic distinction from the four plasmids identified in this study (Figure 5C). We have also performed a BLASTn search using the genomes of the four plasmids identified in this study to determine the closest plasmids to them in the GenBank (Supplementary Figures 4A–D). For each plasmid, the top four plasmids with the highest sequence similarity were selected for an OrthoVenn-based orthologous gene clusters determination. The plasmid XtKm15_P1 shared 30 proteins with the *X. albilineans* plasmids GPE PC73 and pXaFJ1, *X. hortorum* pv. *pelargonii* plasmid CFBP2533_p47 and a *Xanthomonas* sp. CPBF 424 plasmid 2 (Supplementary Figure 4A); while the plasmid XtKm15_P2 had 28 shared proteins with the *Aminobacter* sp. plasmid pBAM1, *Cupriavidus necator* plasmid pENH91, *Delftia acidovorans* plasmid pNB8c and *Diaphorobacter* sp. plasmid pDCNB as shown in Supplementary Figure 4B. As for the plasmid XtLr8_P1, 37 shared proteins were detected among the *Yersinia pestis* plasmid pIP1203, *X. vesicatoria* plasmid pLM159.2, as well as the two plasmids pAKD1 and pSN1104-59 from uncultured bacteria (Supplementary Figure 4C). The plasmid XtLr8_P2 shared 21 proteins with the *X. hortorum* pv. *gardneri* plasmid pICMP7383.2, *X. hortorum* pv. *pelargonii* plasmid CFBP2533_p47, *X. hortorum* plasmid pB07007 and a *Xanthomonas* sp. CPBF 424 plasmid 2 (Supplementary Figure 4D).

### Type III Secretion System of *X. translucens*

The *X. translucens* genomes were evaluated for potential variations in their secretion systems and the corresponding genes. Significant variations were found among the 14 *X. translucens* dataset in their membrane fusion (*hlyD*) and ABC transporter (*hlyB*) genes where these genes were lacking in the *X. translucens* pv. *translucens* strains XtKm8 and XtKm34 but present in XtKm9 and the reference strain DSM 18974^T^. None of the *X. translucens* pv. *undulosa* strains sequenced in this study had these genes. The strain XtKm9 was more similar to the *X. translucens* pv. *cerealis* strains than to the other *X. translucens* pv. *translucens* strains in the evaluated features. Except for the *hlyD* and *hlyB* genes, all the *X. translucens* pv. *undulosa* strains were similar to each other in the secretion system repertories as shown in Figure 2A. To assess the pathogenicity repertories of *X. translucens* we compared the T3SS features among the 14 dataset. Due to the pivotal role of T3SS in delivering virulence associated effector proteins into host cells any defect in the T3SS will be leading to an attenuated virulence or a complete loss of bacterial pathogenicity. The T3SS in all *X. translucens* strains is encoded by 25 genes from *hpaH* to *hpaD* (>23 kb). The structural organization of the 25 genes in all *X. translucens* was almost identical (Supplementary Figure 5A). We identified six *hpa*, eight *hrp* and eleven *hrc* genes which are conserved in all *X. translucens* strains. The T3SS regulatory genes, *hrpG* and *hrpX*, were positioned inside the *hrp* gene cluster in all *X. translucens*, different from other Xanthomonas species (Supplementary Figure 5A). The structure and arrangement of T3SS in the 14 *X. translucens* dataset were compared against a set of five plant pathogenic xanthomonads representing members from clade I and II of the genus (Supplementary Figure 5B). In *X. translucens* an unknown ORF gene was found between the *hpaT* and *hrcC* loci while the *hpaB* gene was located between the *hrpE* and *hrpG* genes which were different from the other xanthomonads.

### Non-TAL Effectors (Xops) of *X. translucens*

*In silico* analyses revealed that the 14 *X. translucens* strains investigated in this study encode a set of 29–36 non-TAL effectors (Xop) as shown in Table 2. As for the strains sequenced in this study, the number of Xops was 30 in XtFa1, 29 in XtLr8, 31 in XtKm12, 29 in XtKm15, 34 in XtKm8, 36 in XtKm9, and 33 in XtKm34. Furthermore, 29 and 30 Xops were predicted in the genome sequences of the strains LW16 and P3, respectively (Table 2). Among the Xops predicted, 17 Xops, i.e., AvrBs2, XopAA, XopAF1, XopAM, XopAP, XopAV, XopAZ, XopC2, XopF, XopG, XopK, XopN, XopP, XopQ, XopV and XopX as well as XopR (possessing a frameshift mutation) were conserved among all the 14 *X. translucens* dataset. Our results revealed eight core Xops, i.e., AvrBs2, XopF, XopK, XopN, XopP, XopQ, XopX, and XopR in the 14 *X. translucens* dataset while in the previous studies the two effectors XopL and XopZ have also been predicted as the core T3Es. Although two copies of XopL have previously been reported in Xtu 4699 and ICMP 11055 strains we did not find this T3E in our analyses, while three copies of XopL were found in CFBP 2541 instead of previously reported four (Peng et al., 2016; Falahi Charkhabi et al., 2017; Shah et al., 2019). All the 14 *X. translucens* dataset contained multiple copies of AvrBs2 (*n* = 2), XopAF1 (*n* = 1–2), XopAZ (*n* = 2), XopF1 (*n* = 2), XopL (*n* = 0–4), XopP (*n* = 1–3) and XopX (*n* = 3). Several inconsistencies were observed among the results obtained in this study and those reported in the literatures regarding the presence/absence and the copy number of T3Es in *X. translucens* (Peng et al., 2016; Falahi Charkhabi et al., 2017; Shah et al., 2019). A number of Xops were detected in the sequences of reference *X. translucens* strains in our analyses which have not previously been reported in their respective strains. For instance, in the strains Xtu 4699 and ICMP 11055 XopAV (*n* = 1) and XopAZ (*n* = 2), in the strain NXtc01 XopAV (*n* = 2) and XopZ (two copies instead of one), in CFBP 2541 AvrXccA1 (*n* = 1), XopAV (*n* = 2) and XopAZ (*n* = 2) and in DSM 18974^T^ XopAJ (*n* = 1), XopAL1 (*n* = 1), XopAV (*n* = 2), XopAZ (*n* = 2), XopE5 (*n* = 1) and XopM (*n* = 1) were different in our analyses from those reported in the literatures (Table 2).

**TABLE 2:** Predicted type III effectors (T3Es) in the seven *Xanthomonas translucens* strains sequenced in this study as well as the seven reference strains obtained from the NCBI GenBank.

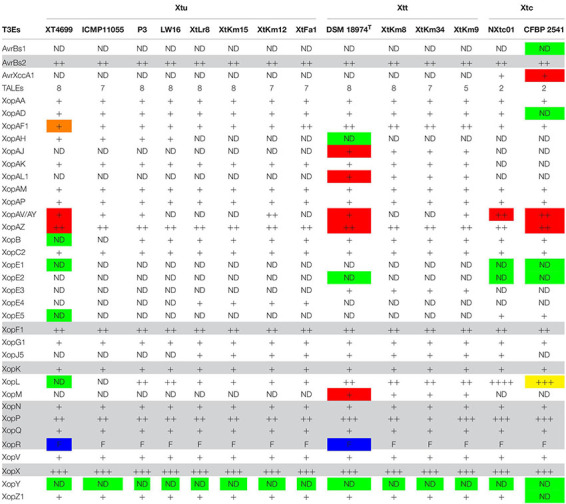

*Hypothetical T3Es were predicted using BLASTn and BLASTp. The annotated T3E sequences were obtained from the NCBI GenBank, https://www.uniprot.org/, http://xanthomonas.org/ and EuroXanth DokuWiki. The number of T3Es varied between 29 and 36 while eight genes were identified as core T3Es (highlighted in gray). The number of plusses (+) indicates the copy number of T3Es detected in each genome; ND, not detected; F, the gene harbors a frameshift mutation. Green highlight: T3Es have been reported previously but not found in our analyses. Red highlight: T3Es have not been reported previously but found in our analyses. Blue highlight: T3Es have been reported previously in full length but found to be frameshifted in our analyses. Orange highlight: two copies of T3Es have been reported previously but we found a single copy. Yellow highlight: T3Es have been reported previously in four copies but we found in three copies.*

On the other hand, a number of Xops have previously been reported in certain reference strains but have not been detected in our analyses. For instance, AvrBs1, XopAD, XopE1, XopE2 and XopZ1 in CFBP 2541; XopE1 and XopE2 in NXtc01; XopAH and XopE2 in DSM 18974^T^ and XopB, XopE1, XopE5, XopG1 and XopL in Xtu 4699 and ICMP 11055 were absent in our analyses. Furthermore, in Xtu 4699, two copies of XopAF1 have previously been reported while only a single copy was found in our analyses. A single copy of XopAF1 was found in all *X. translucens* pv. *undulosa* and *X. translucens* pv. *cerealis* strains except for the strain XtFa1 which had two copies, while all *X. translucens* pv. *translucens* strains carried two copies of XopAF1. AvrXccA1 was detected only in *X. translucens* pv. *cerealis* strains while XopAD was predicted in all 14 *X. translucens* dataset except for CFBP 2541. Further, XopAH has not been detected in any of seven strains sequenced in this study. XopAJ, XopAL1 XopE3 and XopM were found in all *X. translucens* pv. *translucens* strains but not in *X. translucens* pv. *undulosa* and *X. translucens* pv. *cerealis* strains (Table 2), indicating their host specificity nature. Considering the seven reference strains, inconsistencies between the data reported in the literature and those obtained in this study could be due to the continuous up-gradation in the T3E databases.

### TALE Diversity in *X. translucens*

**FIGURE 6:**
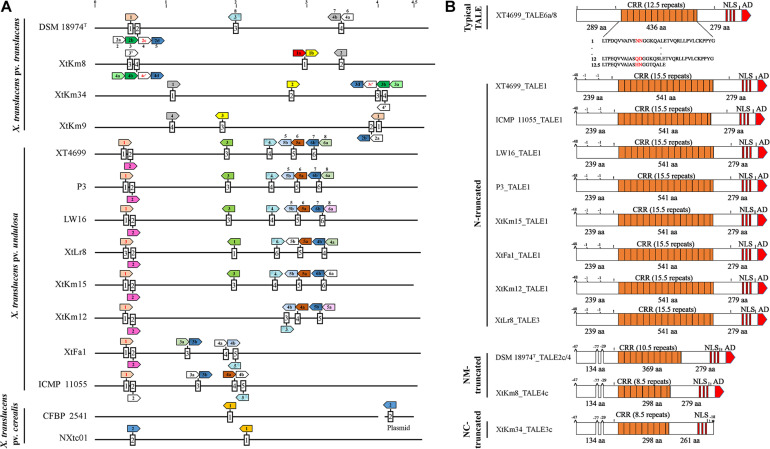
TALE (transcription activator-like effector) repertories of the seven *Xanthomonas translucens* strains sequenced in this study in comparison to the seven reference sequences obtained from the NCBI GenBank. **(A)** A linearized plot of the chromosomes of 14 genomes starting from DNA gyrase subunit B (*gyrB*) gene to indicate the distribution and localization of TALE genes in each strain. Each box represents a TALE locus where TALEs that had identity at the 12th and 13th codons (Zero to three RVDs difference) are indicated by colored arrows while pseudogenes are indicated by red fonts inside the arrows. Names of the previously characterized TALEs are indicated above the corresponding arrow. TALEs having RVDs ≤ 9 are indicated by an apostrophe. The remaining TALEs (≥four RVD differences), unique to each strain are not colored. Scale at top of chromosomes in MB. **(B)** Schematic maps of truncated tal genes where Xtu 4699_TALE6a/8 was used as a reference gene at top. All other TALEs have short N-terminus while XtKm34_TALE3c have deletion of amino acids (aa) both at N- and C-termini. The number of RVDs in each TALE is specified. Symbols shown above the TALE horizontal line (|) *Bam*HI site (!) *Sph*I site, ^ deletion of 29 to 77 aa at start and middle of N-termini, ′ means deletion of single aa and * denote missing activation domain at the said position comparing with reference TALE. Length of CRR, N- and C- termini are displayed in aa.

Complete genome sequence-based investigations have determined the TALE repertories of the seven strains sequenced in this study where the number of TALEs in the strains XtKm8 = 8, XtKm9 = 5, XtKm34 = 7, XtKm12 = 7, XtKm15 = 8, XtFa1 = 7 and XtLr8 = 8 (Supplementary Table 1). In most of the TALE genes, conserved *Bam*HI and/or *Sph*I sites at either N- and/or C-terminus were missing. TALEs of XtKm8 encoded proteins with 7 to 22 RVDs, while TALEs of XtKm9 encoded 12 to 17 RVDs, and TALEs of XtKm34 encoded 7 to 19 RVDs. TALEs of the strains XtKm15, XtLr8, and XtKm12 encoded 14–18, 12–18, and 15–18 RVDs respectively, while the TALEs of XtFa1 encoded 13 to 18 RVDs. A schematic map of *tal* genes in all 14 *X. translucens* strains is illustrated in Figure 6A where all chromosomes are linearized starting from DNA gyrase subunit B (*gyrB*) gene. The *tal* genes were distributed among four loci in *X. translucens* pv. *translucens*, while in five to six loci in *X. translucens* pv. *undulosa* and in two loci in *X. translucens* pv. *cerealis*. In the *X. translucens* pv. *translucens* strains DSM 18974^T^ and XtKm8 two loci consisted of a single gene, one locus consisted of two *tal* genes and the other locus consisted of four, all oriented in the same direction. However, in the strains XtKm9 and XtKm34 three loci consisted of a single gene, one locus in XtKm34 consisted of four and one locus in XtKm9 consisted of two genes. In *X. translucens* pv. *cerealis* both loci comprised of a single *tal* gene entirely conserved between the two strains, while in the strain CFBP 2541 one of the *tal* genes was located on an endogenous plasmid. In *X. translucens* pv. *undulosa* strains Xtu 4699, P3, LW16, XtLr8 and XtKm15, four loci possessed single *tal* gene whereas in the strains XtKm12, XtFa1 and ICMP 11055 three loci had single genes. The remaining two loci in all the *X. translucens* pv. *undulosa* strains had two *tal* genes oriented in the same direction (Figure 6A). The number of *tal* genes in the seven *X. translucens* strains sequenced in this study was also confirmed using southern blot technique as detailed previously (Khojasteh et al., 2020).

In addition, a number of truncated *tal* genes were predicted among the 14 *X. translucens* dataset. For instance, the TALEs XtKm8_tal4c, XtLr8_tal3, DSM 18974^T^_tal2c and tal1 in the strains XtFa1, XtKm12, XtKm15, Xtu 4699, ICMP 11055, LW16 and P3 were distinct from the other TALE genes having shortened N-terminus while XtKm34_tal3c have shortened both N- and C-termini (Figure 6B). Xtu 4699 contained eight *tal* genes on six loci where TALE6a/8 which is known as virulent factor contributing to bacterial leaf streak development in wheat was used as a reference gene to determine pseudogenes (Figure 6B). Some *tal* genes including Xtu 4699_tal1, ICMP 11055_tal1, LW16_tal1, P3_tal1, XtKm15_tal1, XtFa1_tal1, XtKm12_tal1 and XtLr8_tal3 has 48 amino acid (aa) deletion at the start of N- terminus and also lack classically conserved *Bam*HI site. Single aa deletions were also found in all these *tal* genes at the mid of N-terminus. Some other *tal* genes, e.g., DSM 18974^T^ _tal2c/4, XtKm8_tal4c and XtKm34_tal3c had 48 aa deletions at start and 29 to 77 aa at middle of N-terminus. XtKm34_tal3c carried a premature stop codon, probably encoding a protein with a C-terminal truncation of 18 aa, leading to deletion of activation domain (Figure 6B). All truncated *tal* genes lack classically conserved *Bam*HI site at N-terminus and *Sph*I site at C-terminus of some genes.

TALEs of all 14 *X. translucens* strains composed of both 34 and 35 amino acid repeat types except for the last repeat of each TALE. In each repeat, 12th and 13th amino acids (termed RVD) comprised of some unusual RVDs, i.e., KG, QD, YK, YD, NF, GI, KI and Y^∗^. These RVDs have rarely been found in xanthomonads. In the strain XtKm8, two unique RVDs HE and YI were identified in the TALE XtKm8_TALE1a that have not previously been reported. Among the 14 *X. translucens* strains TALEs were grouped into 16 classes, each class consisting of perfectly or nearly conserved TALEs (up to three variations in RVDs) with the exception of the classes 2, 8, 9, 12, and 16. Only one TALE in class 12 (XtFa1_TALE4a) and class 8 (XtKm9_TALE2a), two TALEs in class 2 (XtKm9_TALE1 and DSM 18974^T^ _TALE1), three in class 9 (ICMP 11055_TALE2, XtKm8_TALE4a and XtKm34_TALE3a) and almost all of class 16 were found distinct from the remaining TALEs in the class (Supplementary Table 1).

**FIGURE 7:**
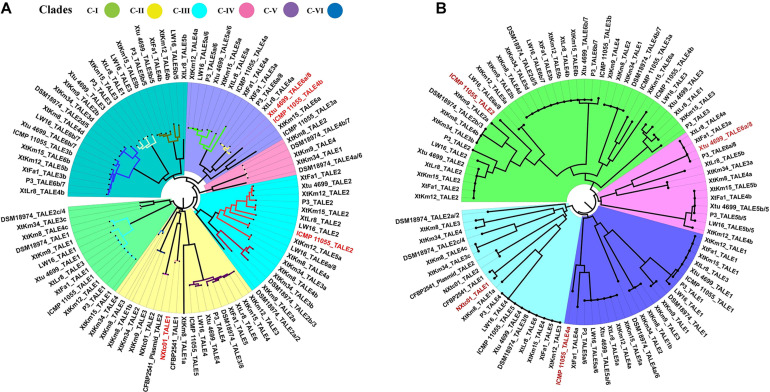
Phylogenetic tree based on TALEs-CRR **(A)** and RVDs **(B)** in the seven *Xanthomonas translucens* strains sequenced in this study as well as the seven reference sequences obtained from the NCBI GenBank. TALEs were designated numerically according to the locus order (Figure 6A). Former TALE names are pointed out in the tree with slash, while red font indicates virulence associated TALEs. **(A)** DisTAL was used to construct a Neighbor-joining tree for a set of 93 TALE sequences from 14 *X. translucens* strains. TALEs were classified into six major clades as block shaded in different colors. TALE classes (Supplementary Table 1) are represented with colored nodes with the exception of class 16 distributed among all clades. **(B)** FuncTAL was used to build a Neighbor-joining tree for a set of 93 TALE sequences based on TALE-RVDs. TALEs were classified into four major groups (16 classes; Supplementary Table 1) as block shaded in different colors.

Four *X. translucens* TALEs have previously been reported to have contribution to virulence and host susceptibility on wheat plants. All these four TALEs were detected in our dataset and were grouped into separate classes where NXtc01_TALE1 was in class 6, ICMP 11055_TALE2 was in class 9, Xtu 4699_TALE6a/8 was in class 11, and ICMP 11055_TALE4b was in class 16. The Xtu 4699_TALE6a/8 encodes the major virulence determinant for Xtu 4699 whose function is to promote disease susceptibility by targeting host gene TaNCED-5BS that encodes 9-cis-epoxycarotenoid dioxygenase (Peng et al., 2019). The class 11 that contains this TALE comprised of perfectly identical four TALEs of *X. translucens* pv. *undulosa* including P3_TALE6a/8, Xtu 4699_TALE6a/8, XtFa1_TALE3a, and XtLr8_TALE4a, suggesting their similar functioning in the host plant (Supplementary Table 1). Unexpected similarity in the CRR domain of TALEs between the *X. translucens* pv. *translucens* strains XtKm8, XtKm9 and XtKm34 and those of the *X. translucens* pv. *undulosa* strains XtKm12, XtKm15, XtFa1 and XtLr8 led us to strictly explore their relationship with other *X. translucens* strains (Figures 7A,B). Based on the RVDs-CRR structure of all available complete genomes, *X. translucens* TALEs were classified into five clades (I-V) as shown in Figure 7. Each clade comprised of two or more TALE classes while TALEs of the class 16 that had different TALE-RVDs distributed throughout all clades. In addition, virulence-associated TALEs, i.e., Xtu 4699_tal6a/8, ICMP 11055_tal2, ICMP 11055_tal4b, ICMP 11055 and NXtc01_tal1, were classified in clades II, III and V, respectively. TALEs possessing similar RVDs (TALEs-classes see Supplementary Table 1) grouped on a same node (shown with colored nodes in Figures 7A,B) disseminated either in a single clade (i.e., clade-III) or multiple classes in a single clade, while different TALEs (i.e., class 16) have not been clustered in a single clade suggesting that identical CRRs may encode similar RVDs.

## Discussion

In this study, we have provided the complete genome sequences of seven highly virulent *X. translucens* strains chosen among a set of 57 strains isolated from wheat, barley and ryegrass across the cereal growing areas in Iran. The overall genome structure, phylogenetic position, core *hrp* cluster, non-TALE T3Es and TALE contents of the seven strains were compared with all available complete genomes of *X. translucens* in the NCBI GenBank, i.e., two *X. translucens* pv. *cerealis* strains CFBP 2541 and NXtc01 isolated in the United States and China, respectively, the *X. translucens* pv. *translucens* strain DSM 18974^T^ isolated in the United States and four *X. translucens* pv. *undulosa* strains ICMP 11055, Xtu 4699, P3 and LW16 isolated in Iran and the United States. Despite the temporal and geographic distinctions among the origin of the strains, all the genomes were highly syntenous and their non-TALE T3Es as well as TALE repertoires were highly conserved particularly at pathovar level. The data generated in this study, has doubled the number of publicly available complete genome resources of the bacterial leaf streak pathogen providing a wider framework for the population structure of *X. translucens*.

In the previous study, Khojasteh et al. (2019) have demonstrated high genetic diversity of the bacterial leaf streak pathogens in Iran which is in congruence with the fact that the center of origin of cultivated wheat is determined in the Fertile Crescent overlapping with Iranian Plateau in Karacadağ Mountains in southeast Turkey (Heun et al., 1997; Brandolini et al., 2016). On the other hand, it has been speculated that the new world population of the bacterial leaf streak pathogens has been originated from the Iranian Plateau as indicated by phylogeographic analyses (Khojasteh et al., 2019). Except for the strains ICMP 11055 and NXtc01 which were isolated in Iran and China, respectively, the *X. translucens* strains having available complete genome resources were originated from the United States, narrowing our understanding of the population structure and genomic features of the species. The complete genome sequences provided in this study include a set of taxonomically diverse representatives of the species all isolated from the old world. Comparative genomics and phylogenomics among the entire set of 14 strains provide a comprehensive insight into the global population of *X. translucens*.

The members of *Xanthomonas* encode a typical Hrp-T3SS comprising six hpa (hrp associated), eleven hrc (hrp conserved) and eight hrp genes (Timilsina et al., 2020). Functional analyses have proven the pivotal importance of *X. translucens* T3SS for pathogenicity, induction of HR and delivery of T3Es. However, functional variations were reported for different set of T3SS genes. For instance, mutant of *hrcC* of wheat pathogens NXtc01 and Xtu 4699, and *hrcT* of barley pathogens UPB886, UPB787R and *X. translucens* pv. *hordei* resulted complete loss of the symptom development on host and HR on non-host plants compared to the wild type strain (Peng et al., 2016; Pesce et al., 2017; Shah et al., 2019). In contrast, the *hrcE*, *hrpG* and *hrcR* mutants of grass pathogen Xtg-Xtg29 cannot eliminate disease symptoms completely and colonization is also not effected (Wichmann et al., 2013). Comparison of core *hrp* cluster revealed similar organization in all seven *X. translucens* strains sequenced in this study which was in congruence with their aggressiveness and virulence features described previously (Khojasteh et al., 2020). This study also reveals the non-TALEs/Xop effectors repertoire in *X. translucens* strains in comparison to the other reference genomes of plant pathogenic *Xanthomonas* spp. (Peng et al., 2016; Shah et al., 2019). However, with respect to the previous studies we have noted variations in the copy number, frame-shift mutation and presence or absence of individual Xops (Table 2). Alignment and comparison of genomic data indicate that a core set of T3SEs identified previously is present in the sequenced *X. translucens* genomes but surprisingly two core effectors XopL in Xtu 4699 and ICMP 11055, and XopZ in CFBP 2541 which have previously been reported to present in these strains were not found in our analyses (Table 2). These inconsistencies in the T3SEs might be due to up-gradation of T3Es database implemented in the analyses. The unique T3E repertoire in different pathovars of *X. translucens* and within individual strains might reflect host specificity of the strains to various small grain cereals or specific genotypes of a host (Jacques et al., 2016).

So far, none of the T3Es of *X. translucens* have been functionally characterized nor tested for their contribution to colonization and virulence of the pathogen. TALEs that act like transcription factor inside host nucleus are important virulence factors facilitating the proliferation of the pathogens with the ability to directly bind to the promoter region of the target genes. Comparison of TALEs of the 14 *X. translucens* genomes revealed divergent subfamilies (Figure 7). All *X. translucens* pv. *translucens* strains, i.e., DSM 18974^T^, XtKm8, XtKm9, and XtKm34 share two TALE genes tal4b = tal2 = tal1 = tal4 and tal2d = tal4d = tal3d = tal2b, while the first three strains (DSM 18974^T^, XtKm8 and XtKm9) and last three strains (XtKm8, XtKm9, and XtKm34) share additional one tal gene in each including tal2b = tal4b = tal3b and tal1b = tal2 = tal3. Similarly, one tal of all *X. translucens* (tal2d = tal4d = tal3d = tal2b), and two additional TALEs of DSM 18974^T^ and one of XtKm9 (tal1 = tal1 and tal3/8) are also found in all *X. translucens* pv. *undulosa* strains. Other common TALEs in *X. translucens* pv. *undulosa* strains Xtu 4699, P3, LW16, XtLr8, XtKm15, XtKm12, XtFa1 and ICMP 11055 include tal2 of all strains except ICMP 11055, tal5a and tal4a of all strains excluding XtFa1, tal5b with the exception of XtLr8 and tal4b except for ICMP 11055 and tal3 of some strains (Figure 6A). Some other TALEs were also found common in two or more strains (Figures 6, 7). These identical *tal* genes were acquired prior to pathovars divergence, implying their important role in the pathogenesis of the bacterial leaf streak in small grain cereals. Other *tal* genes which are unique in each strain suggest independent acquisition in the lineage.

A family of TALE variants categorized into two forms, i.e., iTALEs (interfering TALEs) and truncTALEs (truncated TALEs) has been introduced in the previous studies (Ji et al., 2016, 2020; Read et al., 2016). The iTALEs lack C-terminal transcription activation domains due to the introduction of premature stop codon in the coding sequence of the genes, whereas truncTALEs have large deletion of the coding sequence at the 3’ end of the genes (Ji et al., 2020). TALEs comparison of all *X. translucens* strains exhibit one iTALE, i.e., XtKm34_TALE3c possessing truncation of 18 aa at the C-terminal domain due to premature stop codon (Figure 6B), None of the other TALEs had deletion at C-terminus but all TALE variants harbored two conserved internal deletions from 1–77 aa at the N-terminus. Surprisingly, all truncaTALEs retained their CRR, NLS and AD but the variants suffer a large 47–48 aa deletions in the N-terminal region that removes a part of the type III secretion signal. Furthermore, all the truncTALEs also lack classically conserved *Bam*HI site of the N-terminus and found improper distribution of C-terminus *Sph*I site in DSM 18974^T^_TALE2c/4, XtKm8_TALE4c and XtKm34_TALE3c (Figure 6B).

In conclusion, results of the present study revealed a greater diversity in the virulence determinants and pathogenicity repertories among the worldwide population of *X. translucens* than the one that had been described before. More specifically, based on the comparative genomics of the 14 strains we have noted that the strains isolated in Iran are similar to the new world strains in T3E arrangement and non-TAL effectors. However, significant variations were observed in the TALE repertories of the strains. On the other hand, our results suggest that the genomic contents of the bacterial leaf streak pathogens should be further investigated using a pool of strains from all the known hosts of the pathogen, including gramineous weeds in the center of origin of the host crop. Previous studies revealed that presence of plasmids have major impact on metabolic functions and host adaptation (Niu et al., 2015). The presence of plasmids in *Xanthomonas* significantly enhances the tolerance to the stresses of heavy metal ions. Our data could be helpful to further elucidate the biological significance of these plasmids and the adaptive evolution of *X. translucens* pv. *undulosa*. If done, these evaluations will pave the way of searching for new sources of resistance among the wild population of wheat species and will help to find new breeding strategies to develop resistant cultivars. Only future studies based on population genetics, comparative genomics, and pathogenicity assays of a wider collection of strains isolated from different hosts and geographical regions can shed more light on these areas.

## Data Availability Statement

The datasets presented in this study can be found in online repositories. The names of the repository/repositories and accession number(s) can be found below: https://www.ncbi.nlm.nih.gov/genbank/, CP063993, CP063994, CP063995, CP063996, CP063997, CP063998, CP063999, CP064000, CP064001, CP064003, and CP064004.

## Author Contributions

GC and EO conceived and designed the study with assistance from SS and MK. SS and MK carried out the experiments with assistance from PK, LZ, and SM. SS and MK analyzed and interpreted the data with assistance from ST, QW, and ZX. EO prepared the article with assistance from MK, SS, and GC. All the authors revised the final version of the manuscript, while GC and EO acted as the corresponding authors. All authors contributed to the article and approved the submitted version.

## Conflict of Interest

The authors declare that the research was conducted in the absence of any commercial or financial relationships that could be construed as a potential conflict of interest.

## Supplementary Material

Supplementary Figure 1 Circular map of the chromosomal DNA and plasmids of *Xanthomonas translucens* strains sequenced in this study. The figures were generated using Circos software v0.69. The four genomes in the top row are *X. translucens* pv. *undulosa*, the three genomes in the middle row are *X. translucens* pv. *translucens* and the bottom row shows four plasmids in the strains XtLr8 and XtKm15. In each circular DNA map, from the outermost to inner the circles show nucleotide numbering (circle 1), COG annotation of forward (circle 2) and reverse (circle 3) strands, non-coding RNAs (rRNA red, tRNA blue, sRNA green; circle 4), GC content (circle 5) and GC skew (circle 6).

Supplementary Figure 2 Average nucleotide identity (ANI) values among the entire set of *Xanthomonas translucens* including the strains sequenced in this study.

Supplementary Figure 3 Visualization of genomic islands in the chromosomal DNA of the seven *Xanthomonas translucens* strains sequenced in this study as well as the reference strains obtained from the NCBI GenBank using IslandViewer online service.

Supplementary Figure 4 Venn diagrams showing the shared gene families (orthologous clusters) among different sets of plasmids constructed using the OrthoVenn online service. Each diagram indicates the number of shared gene families between one of the four plasmids identified in this study, i.e., XtKm15_P1, XtKm15_P2, XtLr8_P1 and XtLr8_P2 and the four closest plasmids for each of these plasmids in the NCBI GenBank determined by BLASTn.

Supplementary Figure 5 Comparison of the type three secretion system (T3SS) gene clusters in plant pathogenic xanthomonads. **(A)** Organization of the 25 T3SS genes from *hpaH* to *XopF* (about 25 kb) in the 14 *X. translucens* strains evaluated in this study was exactly the same. **(B)** Comparison of the arrangement of T3SS in the 14 *X. translucens* dataset against a set of five plant pathogenic xanthomonads showed that the *X. translucens* strains harbor *hrp* cluster flanked by two *hrp* regulatory genes *hrpX* and *hrpG* which is present outside the *hrp* cluster in all the other evaluated xanthomonads. Furthermore, *hrcL* and *hrcD* were found unique to *X. translucens* which were replaced by *hrpB5* and *hrpD5* in other xanthomonads, respectively.

Supplementary Table 1 Comparison of repeat variable diresidue (RVDs) in the seven *Xanthomonas translucens* strains sequenced in this study as well as the seven reference strains obtained from the NCBI GenBank. TALEs of all 14 *X. translucens* strains were classified into 16 classes where virulence-associated TALEs, i.e., Xtu 4699_TALE6a/8, ICMP 11055_TALE2 and TALE4b and NXtc01_TALE1 are highlighted in yellow, while former TALE names are indicated with slash. Unusual RVDs are shown in red font. Unique RVDs are underlined.

Supplementary Dataset 1 The sequences of all 65 type III effectors (T3Es) retrieved from public databases and include in this study.
