# Supplementary figures and images for: Genomics-Enabled Novel Insight Into the Pathovar-Specific Population Structure of the Bacterial Leaf Streak Pathogen Xanthomonas translucens in Small Grain Cereals
Source: Front Microbiol. 2021 May 28;12:674952. doi: 10.3389/fmicb.2021.674952 (PMC8195340; doi:10.3389/fmicb.2021.674952)

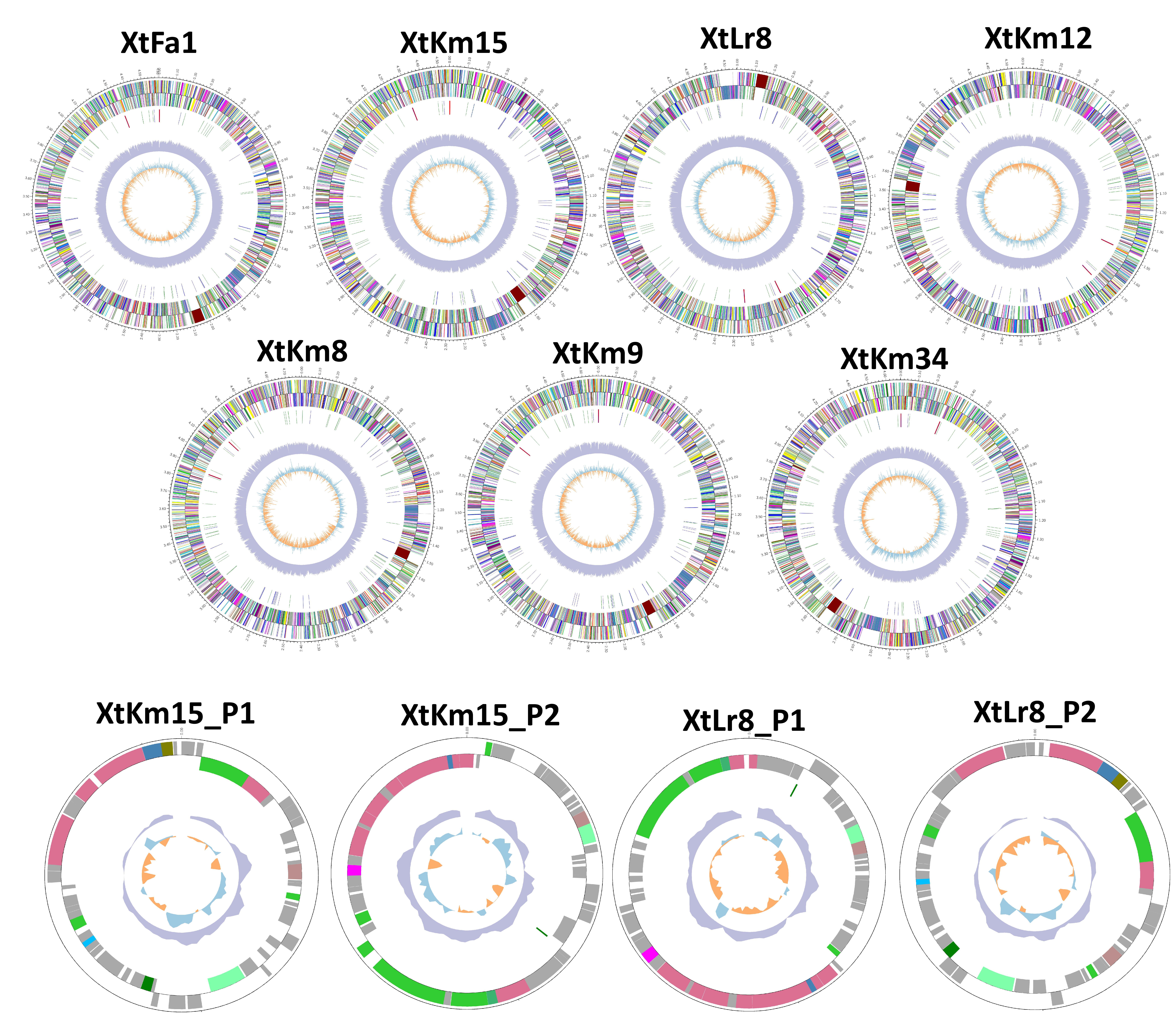

Supplement: Supplementary Figure 1 — Circular map of the chromosomal DNA and plasmids of Xanthomonas translucens strains sequenced in this study. The figures were generated using Circos software v0.69. The four genomes in the top row are X. translucens pv. undulosa, the three genomes in the middle row are X. translucens pv. translucens and the bottom row shows four plasmids in the strains XtLr8 and XtKm15. In each circular DNA map, from the outermost to inner the circles show nucleotide numbering (circle 1), COG annotation of forward (circle 2) and reverse (circle 3) strands, non-coding RNAs (rRNA red, tRNA blue, sRNA green; circle 4), GC content (circle 5) and GC skew (circle 6). [file Image_1.jpg]

DSM 18974

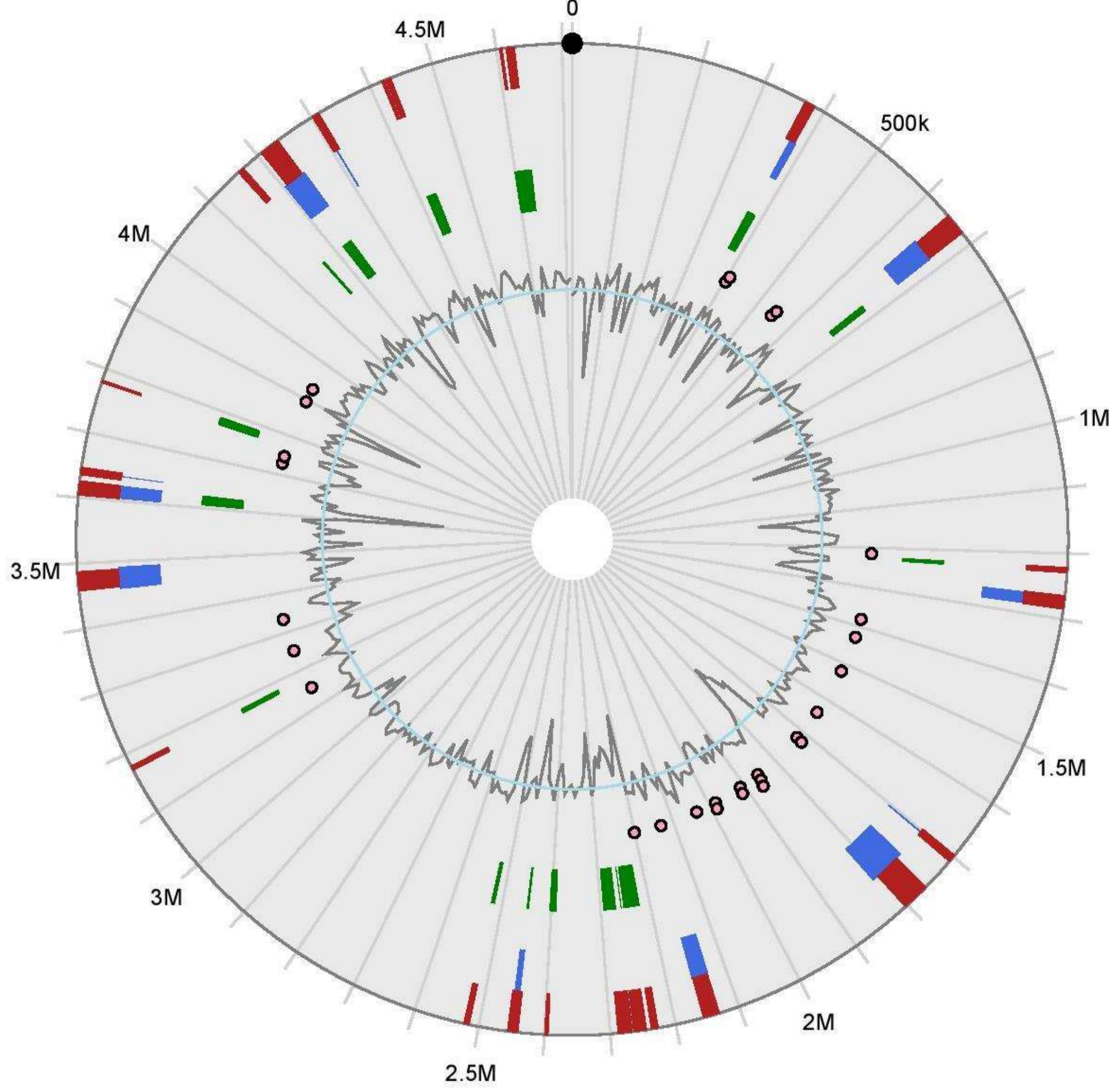

ICMP 11055

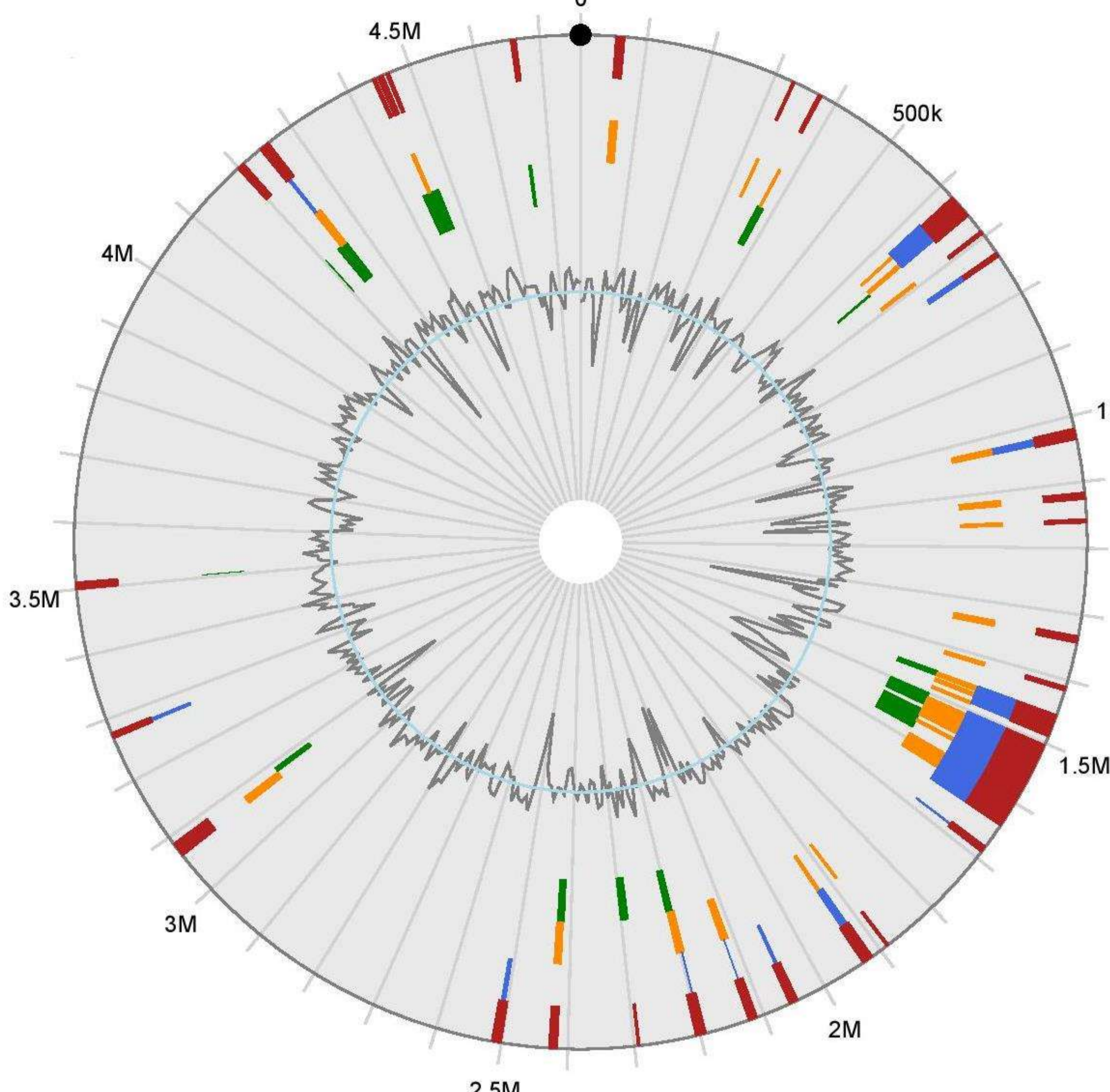

LW16

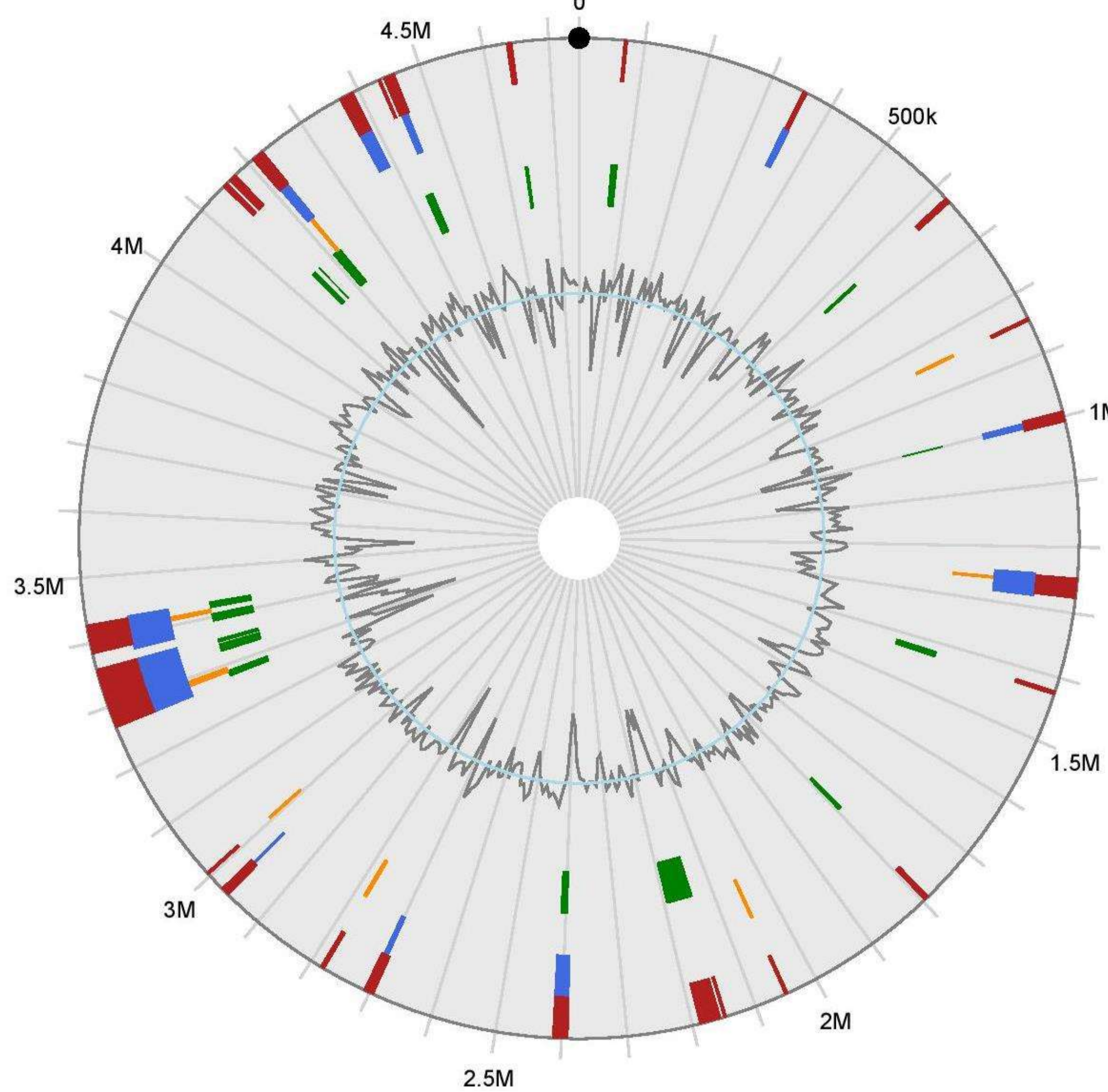

P3

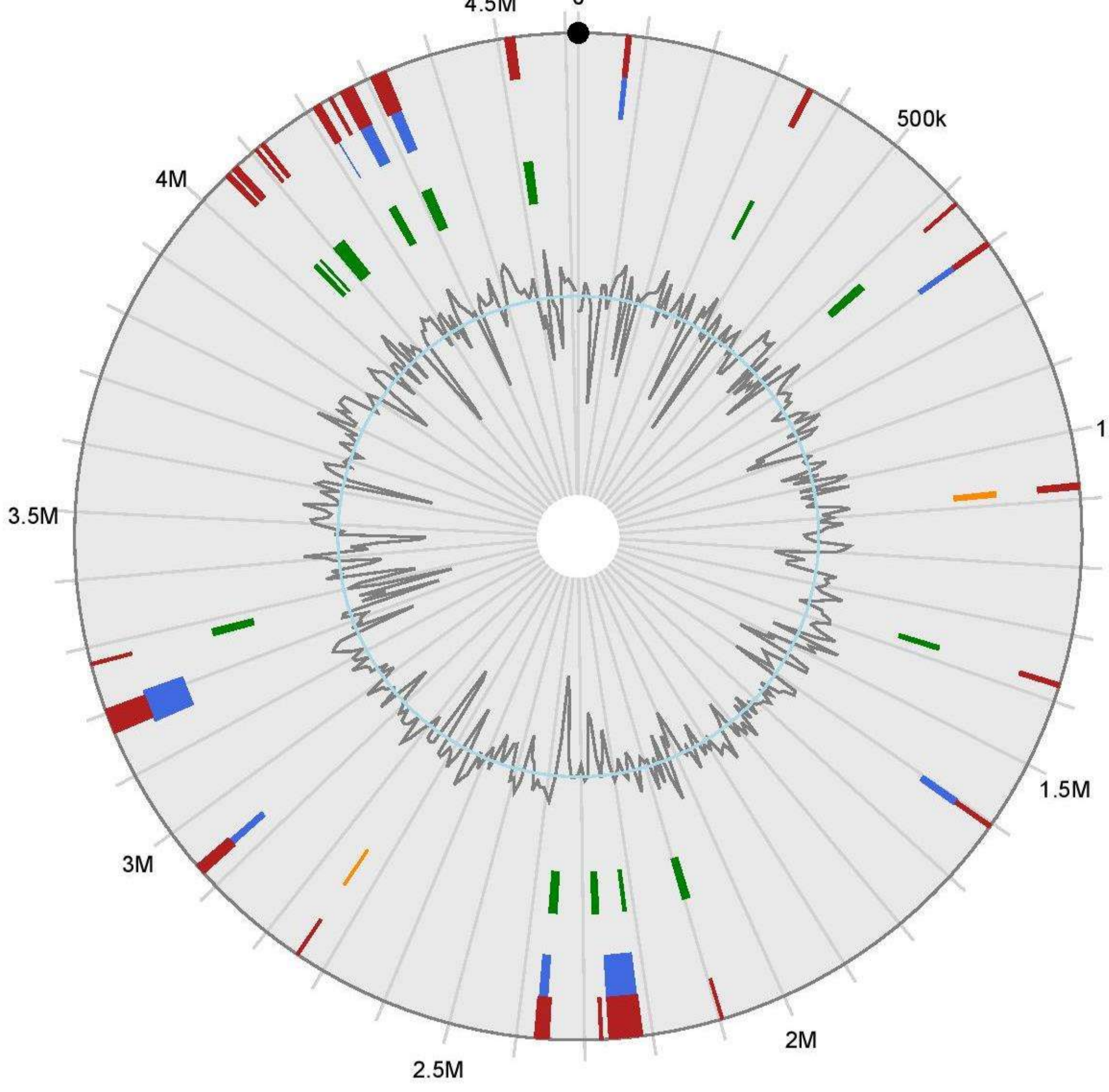

XtFa1

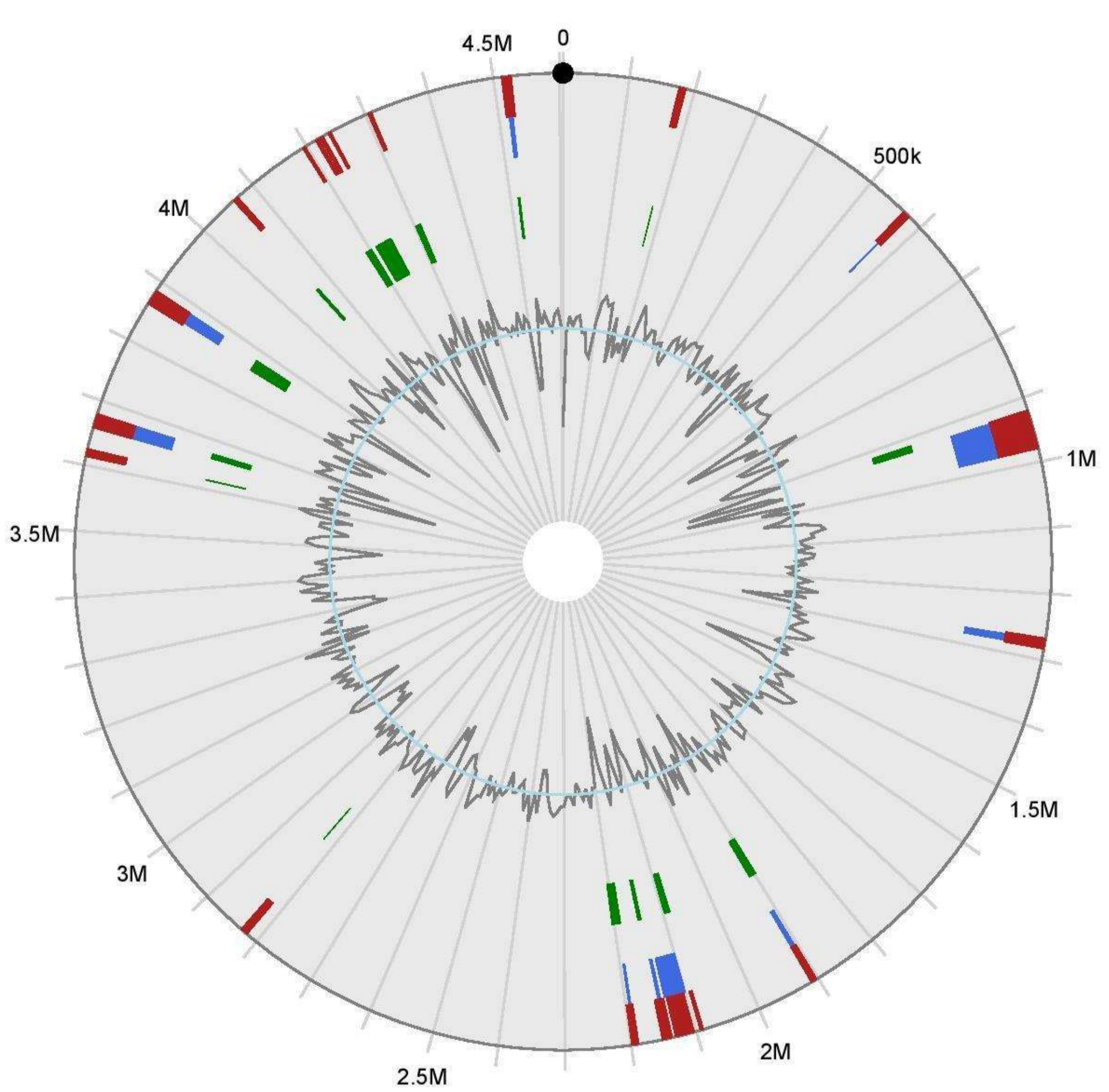

XtKm8

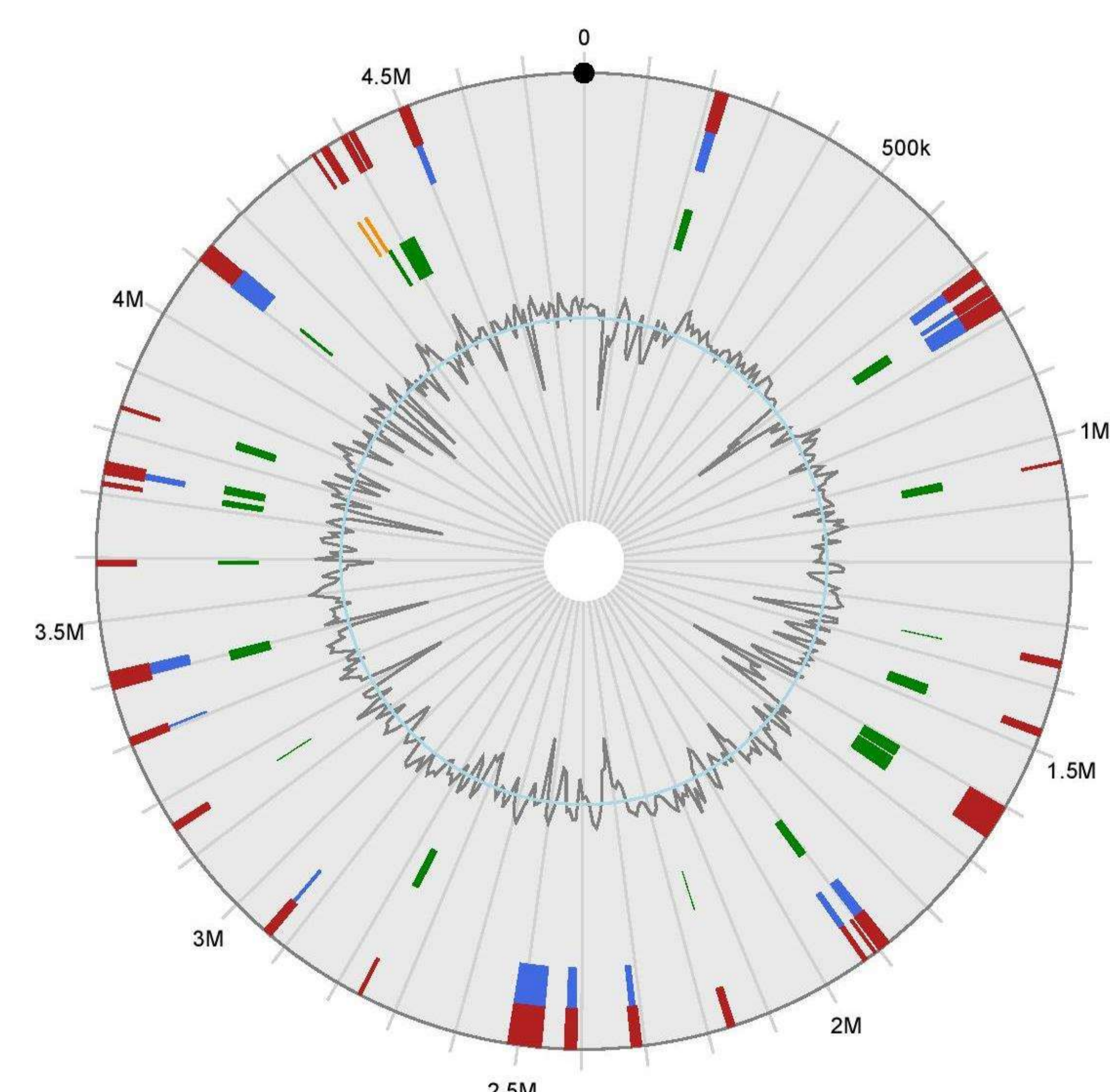

XtKm9

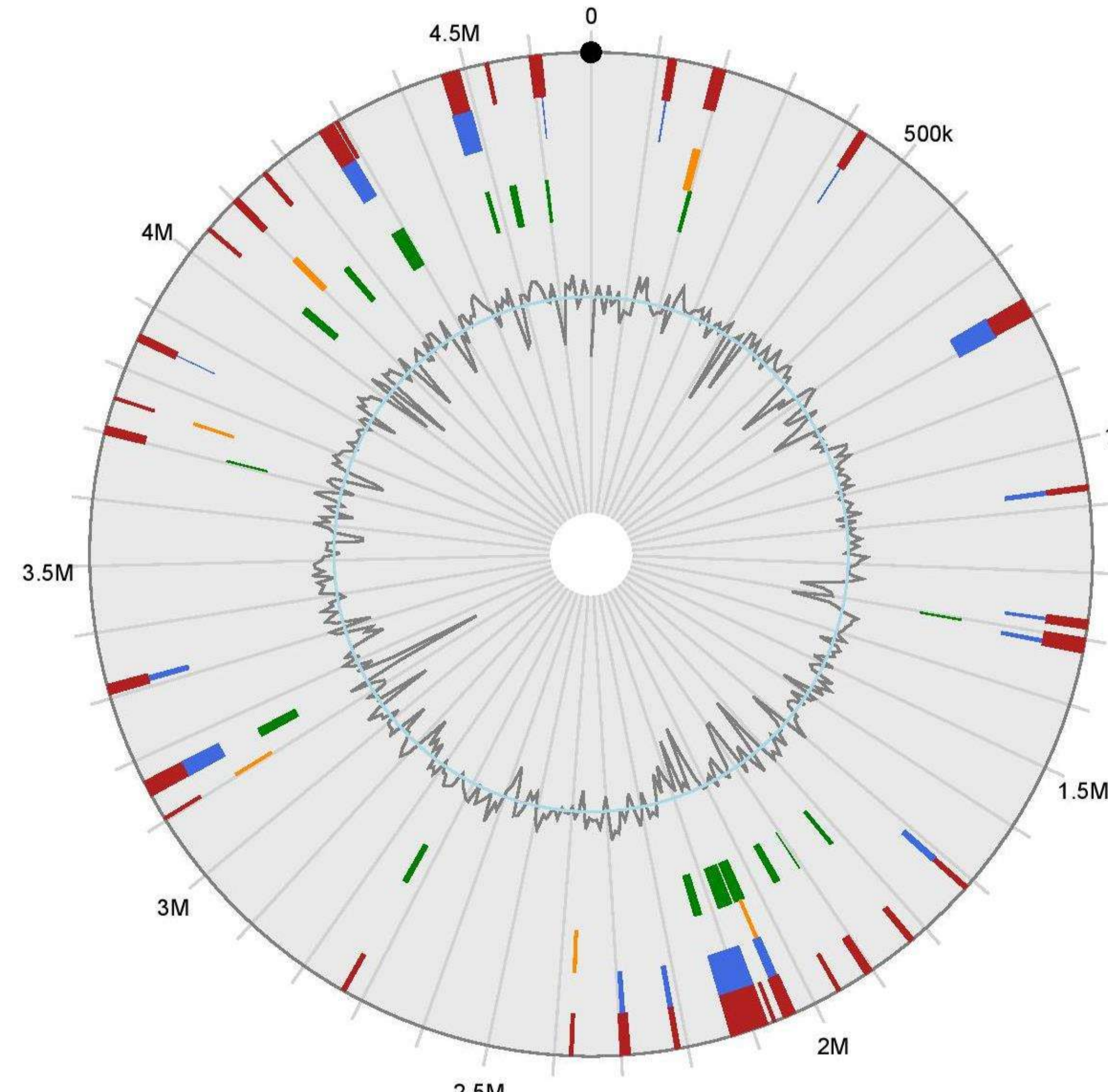

XtKm12

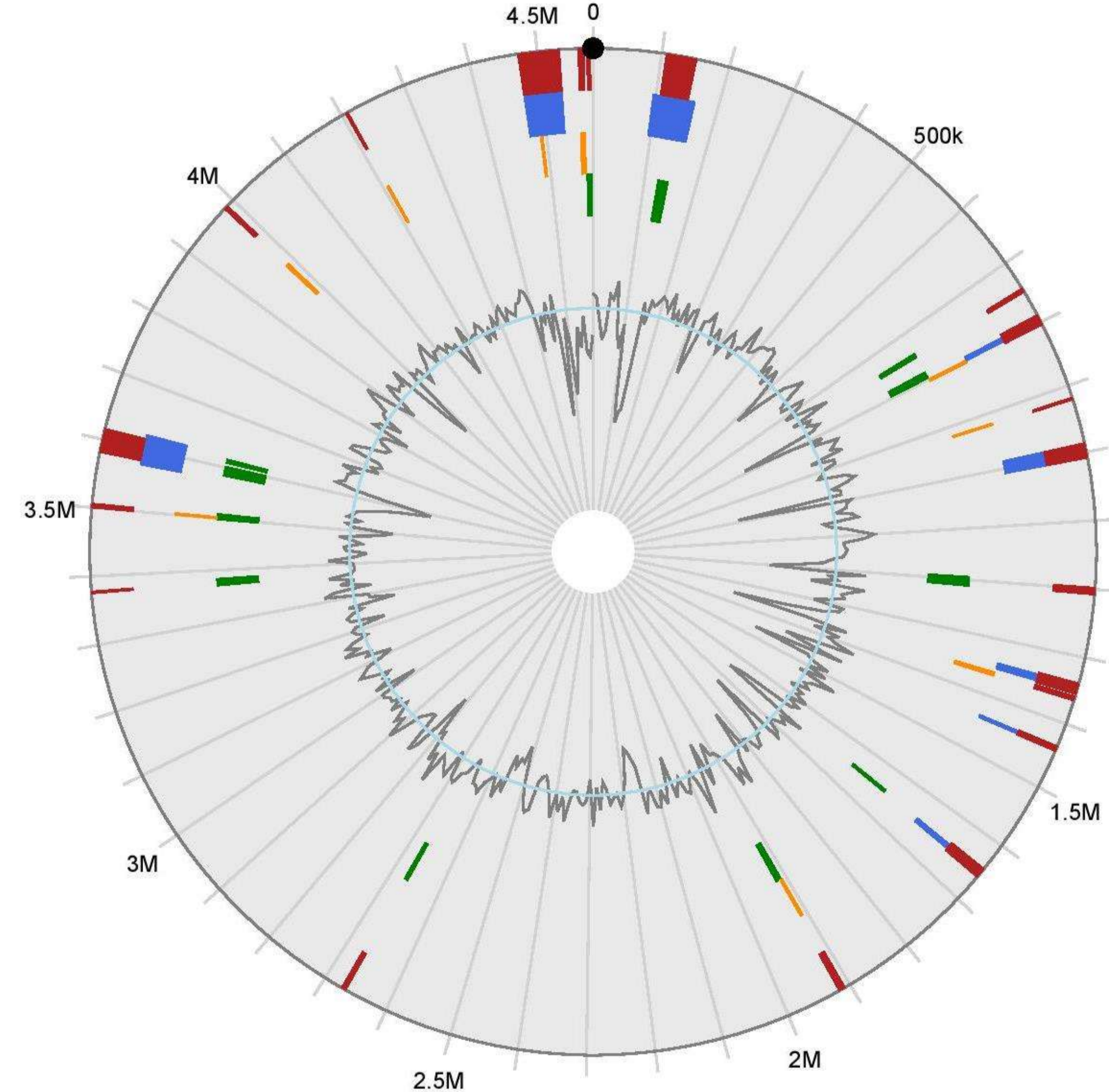

XtKm15

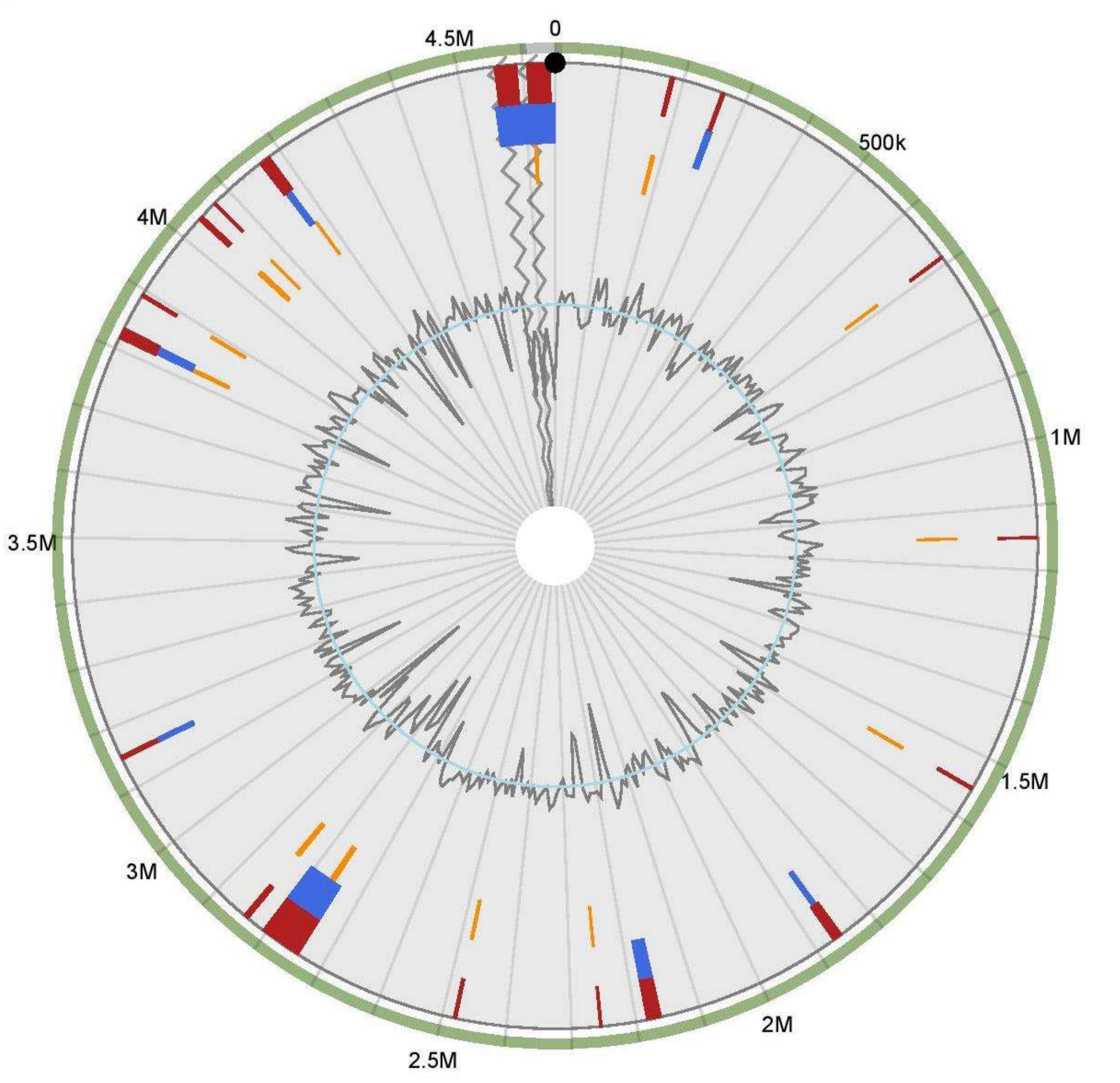

XtKm34

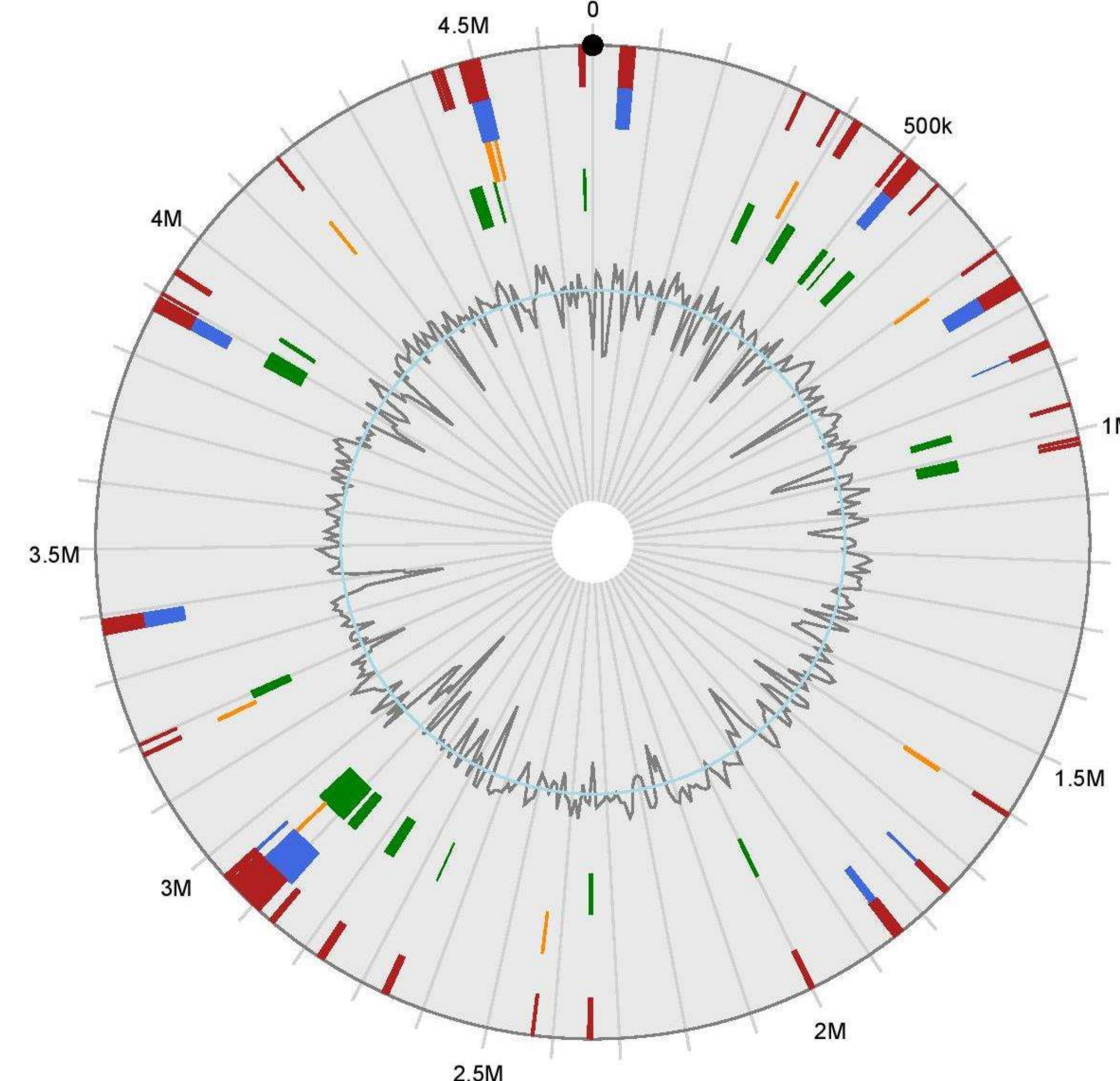

XtLr8

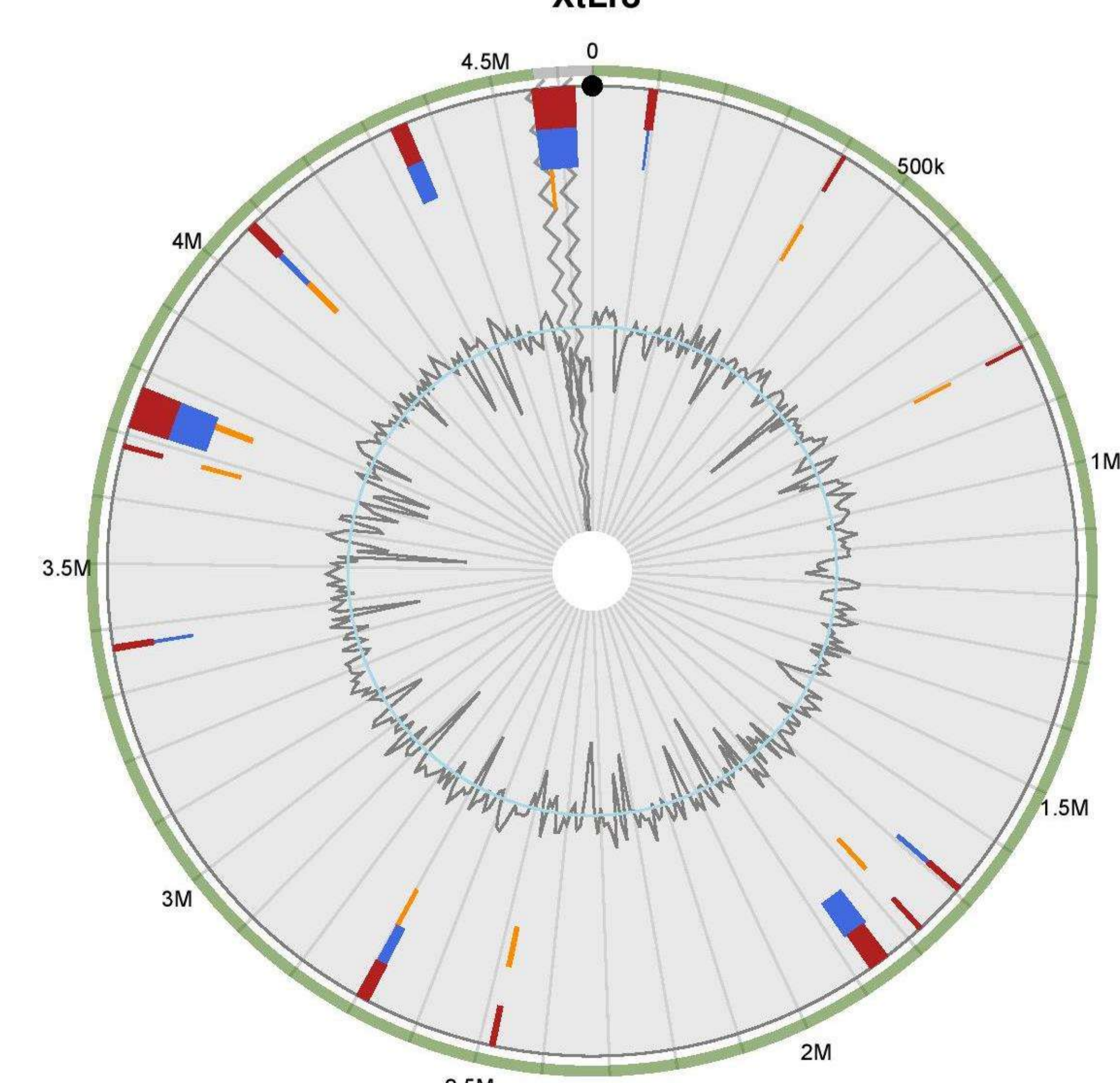

Xtu 4699

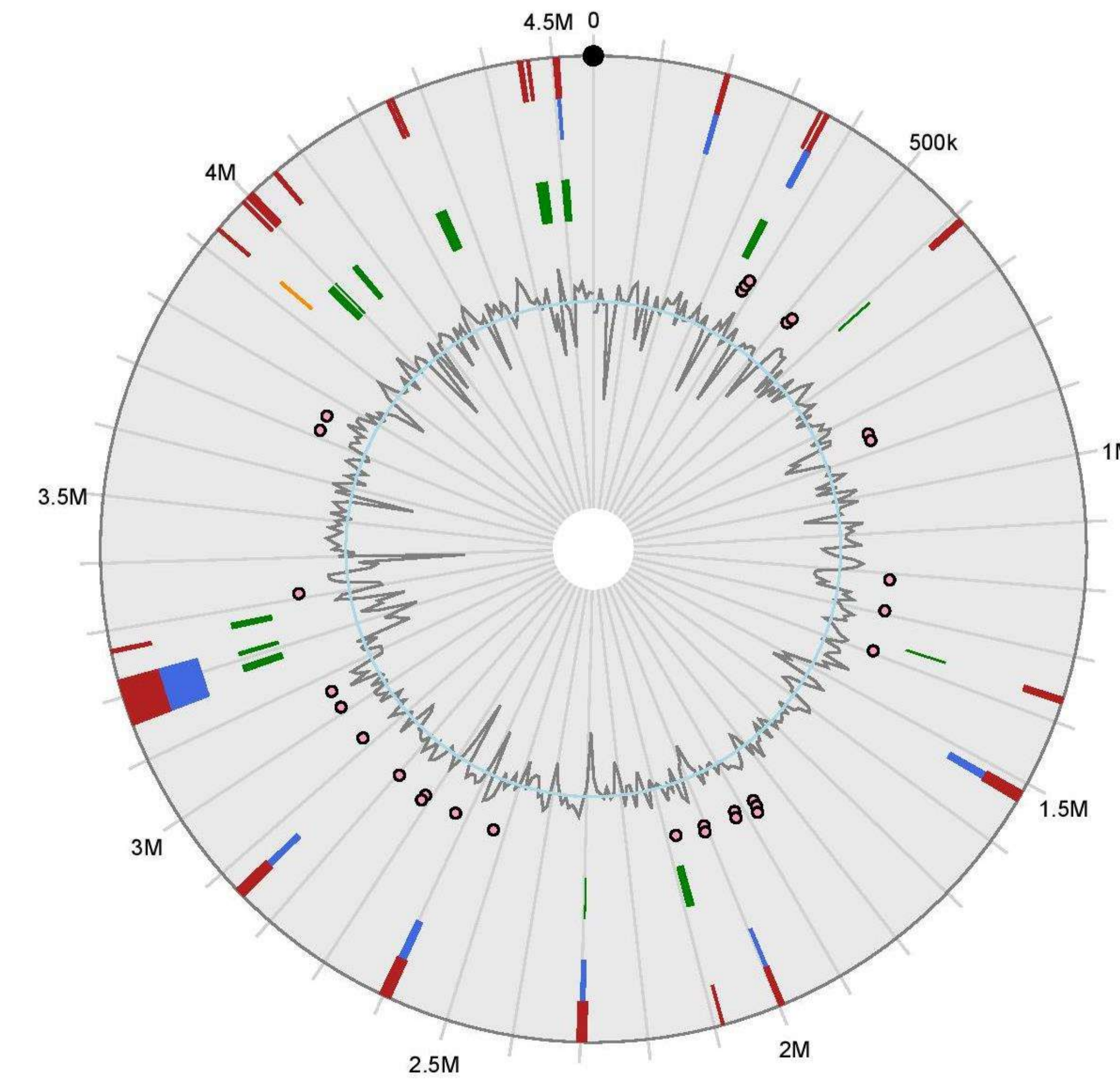

Supplement: Supplementary Figure 3 — Visualization of genomic islands in the chromosomal DNA of the seven Xanthomonas translucens strains sequenced in this study as well as the reference strains obtained from the NCBI GenBank using IslandViewer online service. [file Image_3.pdf]

**A**

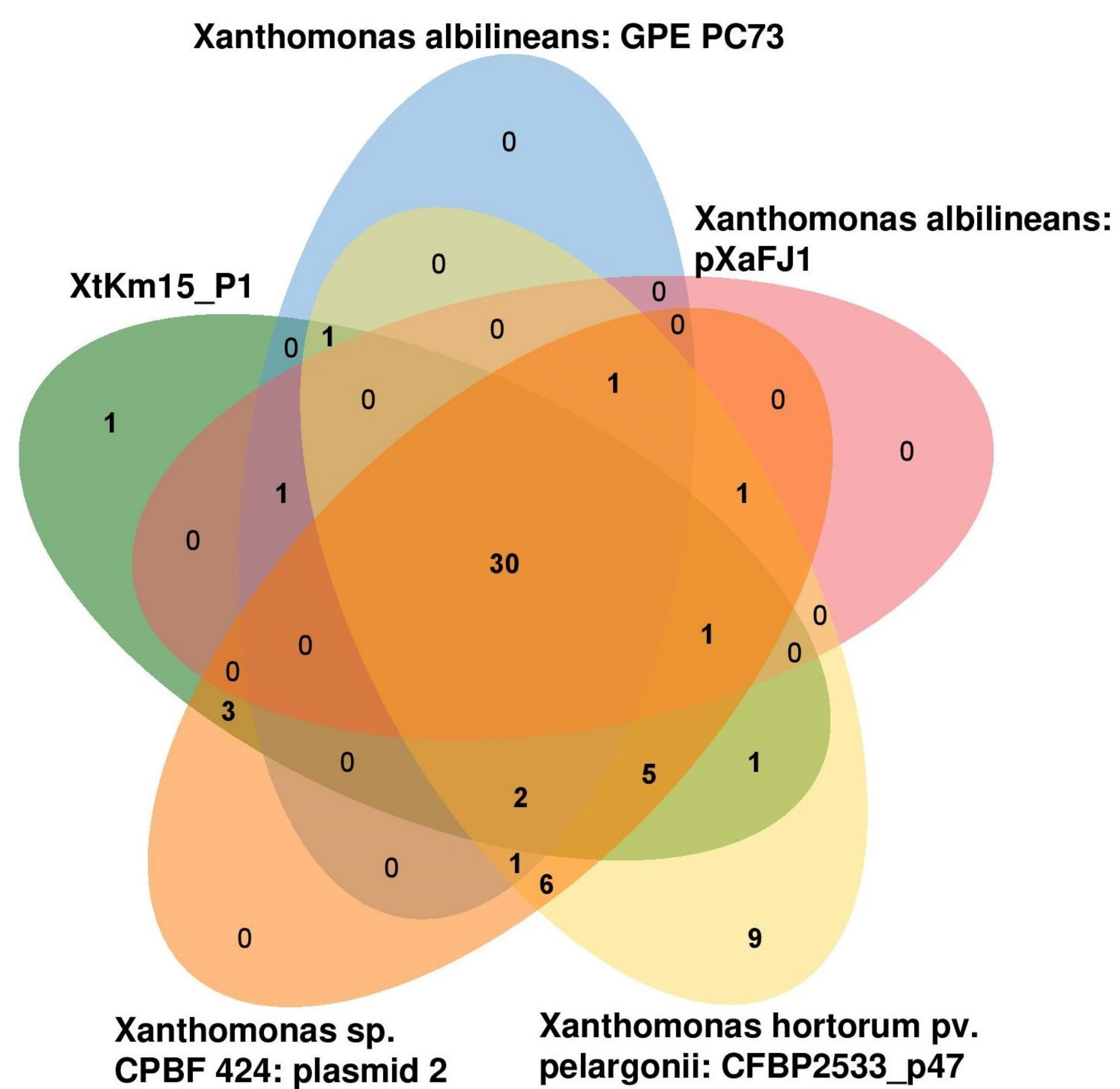

**B**

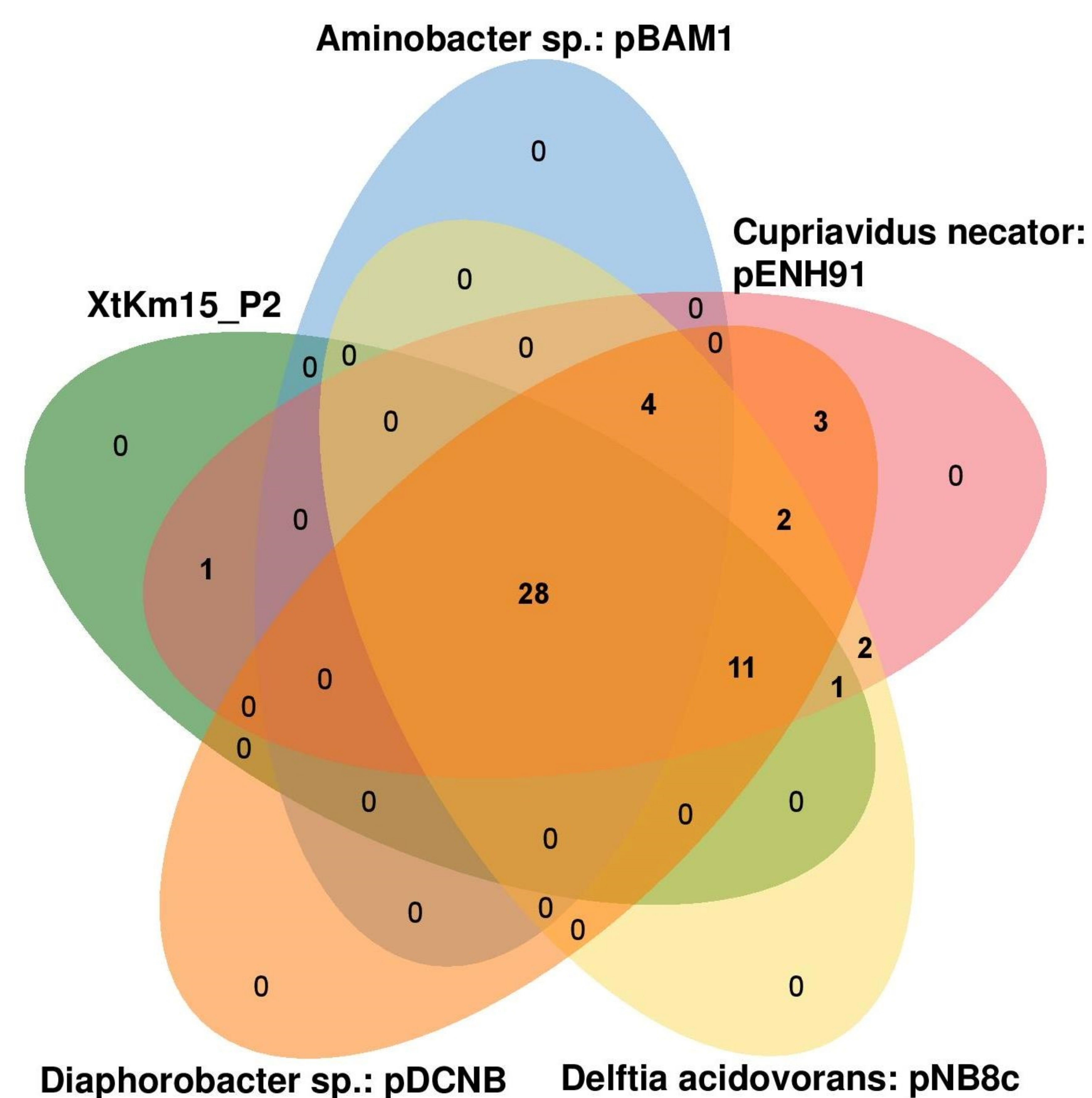

C

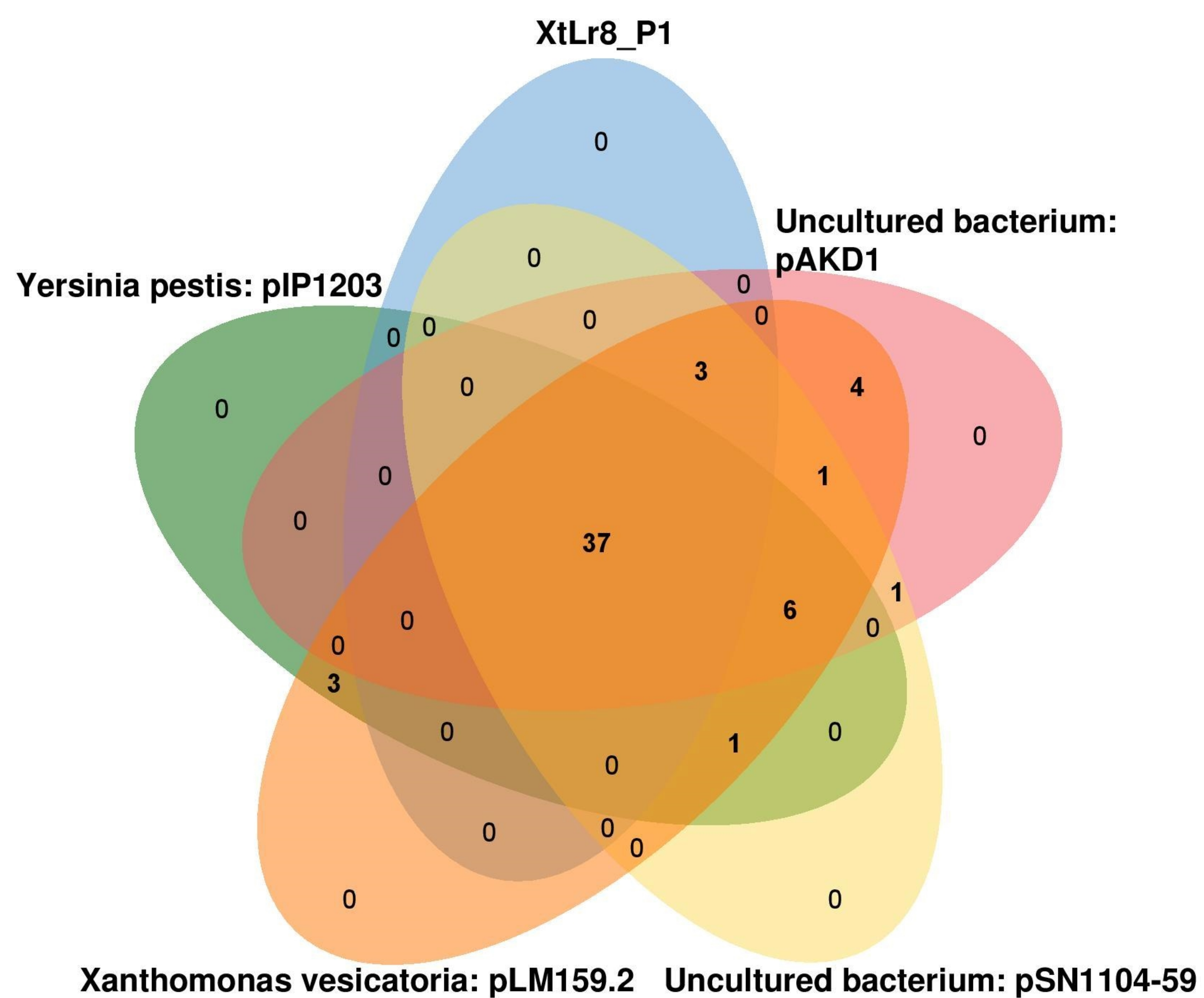

**D**

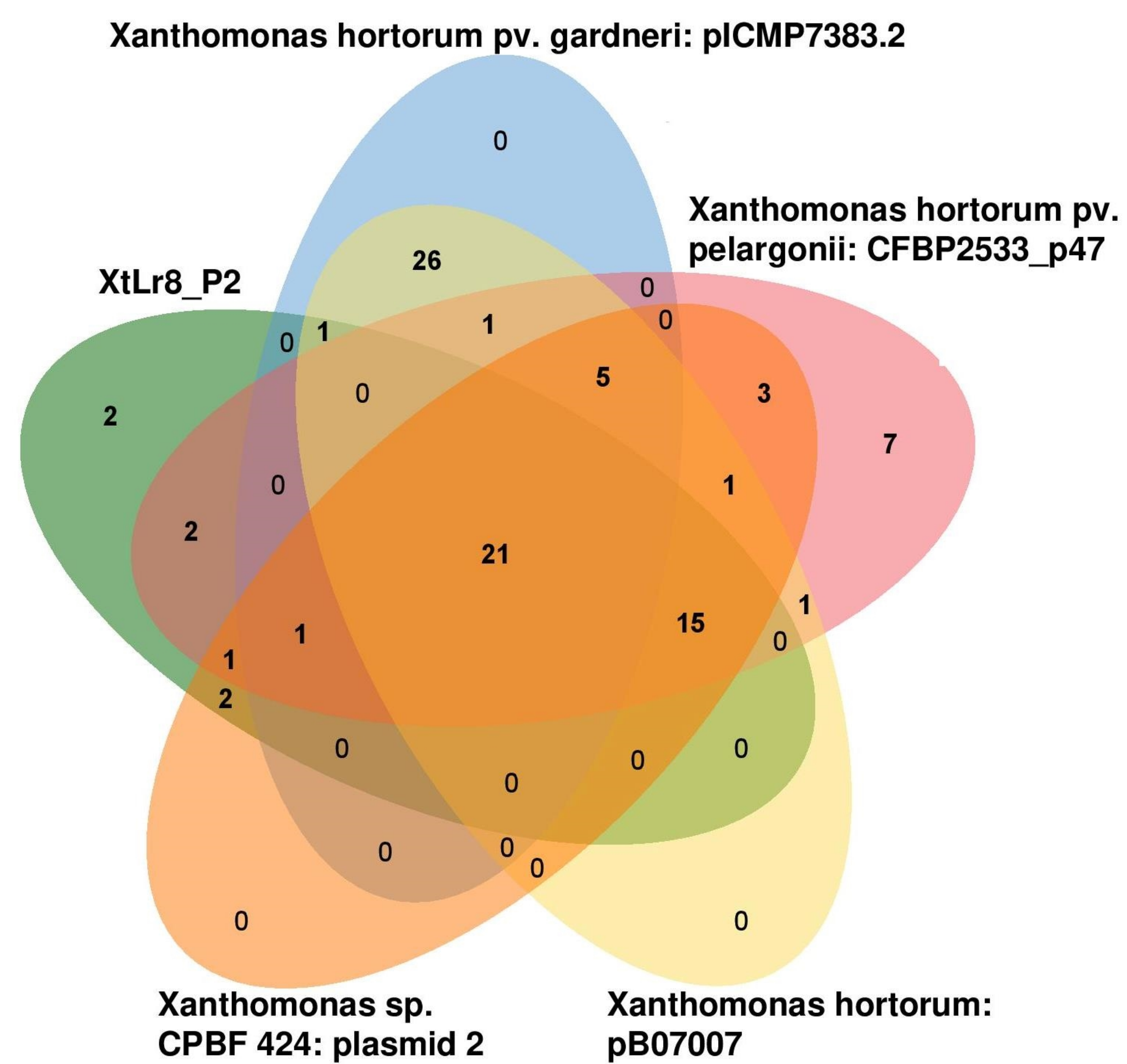

Supplement: Supplementary Figure 4 — Venn diagrams showing the shared gene families (orthologous clusters) among different sets of plasmids constructed using the OrthoVenn online service. Each diagram indicates the number of shared gene families between one of the four plasmids identified in this study, i.e., XtKm15_P1, XtKm15_P2, XtLr8_P1 and XtLr8_P2 and the four closest plasmids for each of these plasmids in the NCBI GenBank determined by BLASTn. [file Image_4.pdf]

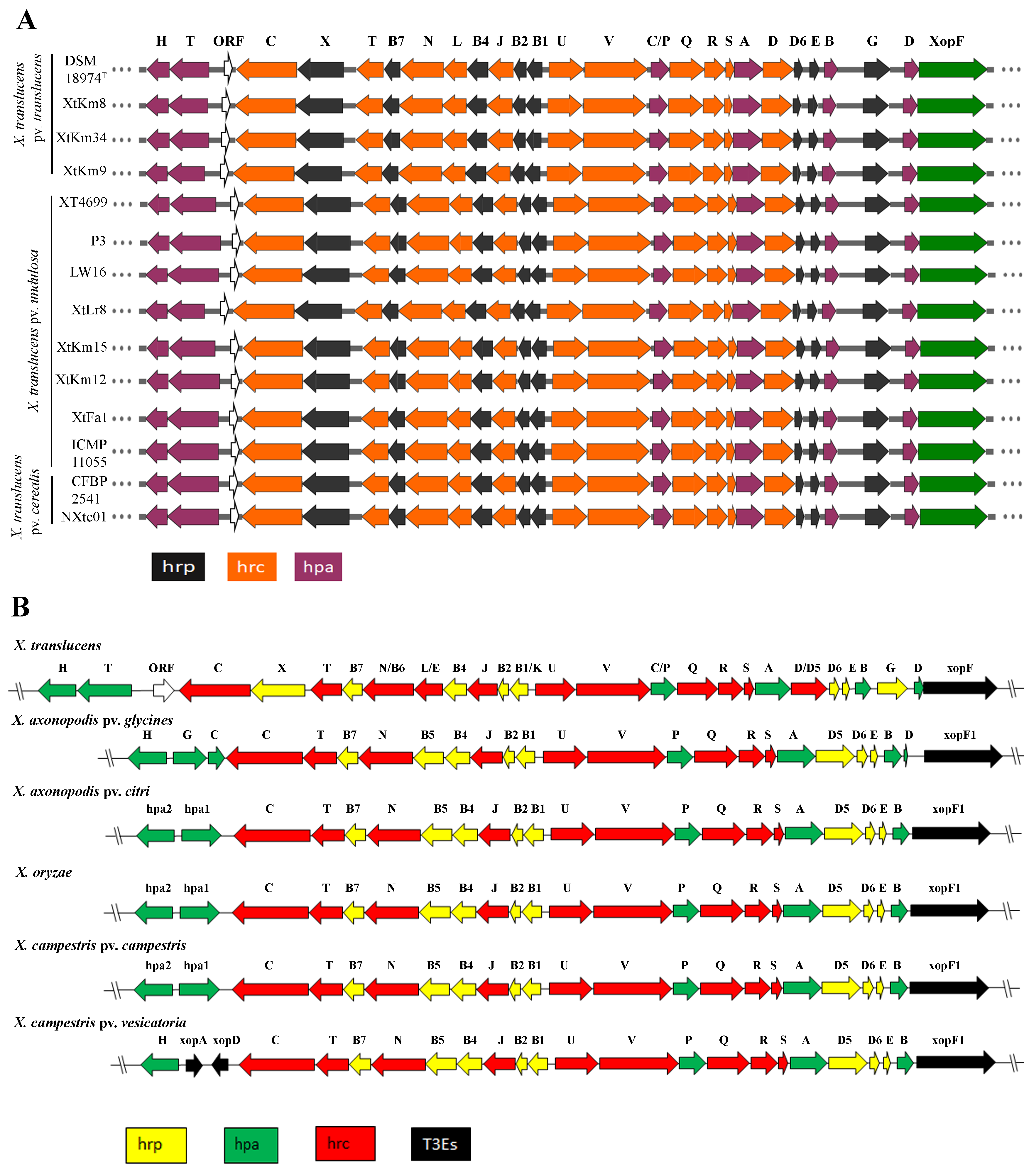

Supplement: Supplementary Figure 5 — Comparison of the type three secretion system (T3SS) gene clusters in plant pathogenic xanthomonads. (A) Organization of the 25 T3SS genes from hpaH to XopF (about 25 kb) in the 14 X. translucens strains evaluated in this study was exactly the same. (B) Comparison of the arrangement of T3SS in the 14 X. translucens dataset against a set of five plant pathogenic xanthomonads showed that the X. translucens strains harbor hrp cluster flanked by two hrp regulatory genes hrpX and hrpG which is present outside the hrp cluster in all the other evaluated xanthomonads. Furthermore, hrcL and hrcD were found unique to X. translucens which were replaced by hrpB5 and hrpD5 in other xanthomonads, respectively. [file Image_5.png]
