# Supplementary material for: Genomics-Enabled Novel Insight Into the Pathovar-Specific Population Structure of the Bacterial Leaf Streak Pathogen Xanthomonas translucens in Small Grain Cereals
Source: Front Microbiol. 2021 May 28;12:674952. doi: 10.3389/fmicb.2021.674952 (PMC8195340; doi:10.3389/fmicb.2021.674952)
Supplement: Supplementary Table 1 — Comparison of repeat variable diresidue (RVDs) in the seven Xanthomonas translucens strains sequenced in this study as well as the seven reference strains obtained from the NCBI GenBank. TALEs of all 14 X. translucens strains were classified into 16 classes where virulence-associated TALEs, i.e., Xtu 4699_TALE6a/8, ICMP 11055_TALE2 and TALE4b and NXtc01_TALE1 are highlighted in yellow, while former TALE names are indicated with slash. Unusual RVDs are shown in red font. Unique RVDs are underlined. [file Table_1.docx]

Table S1: Comparison of repeat variable diresidue (RVDs) in the seven *Xanthomonas translucens* strains sequenced in this study as well as the seven reference strains obtained from the NCBI GenBank. TALEs of all 14 *X. translucens* strains were classified into 16 classes where virulence-associated TALEs i.e. Xtu 4699_TALE6a/8, ICMP 11055_TALE2 and TALE4b and NXtc01_TALE1 are highlighted in yellow, while former TALE names are indicated with slash. Unusual RVDs are shown in red font. Unique RVDs are underlined.

| **TALEs Name** | **RVDs** | |
| --- | --- | --- |
| **Class 1** | **1 2 3 4 5 6 7 8 9 10 11 12 13 14 15 16 17 18 19 20 21 22** | |
| XtKm8_TALE4c | NI HN NG NG ND NK QD NH QD | |
| XtKm34_TALE3c | NI HN NG NG ND NK QD NH QD | |
| **Class 2** | **1 2 3 4 5 6 7 8 9 10 11 12 13 14 15 16 17 18 19 20 21 22** | |
| XtLW16_TALE1 | HD YD NI NG NG NN YK NG HD NG NG ND NG QD NH QD | |
| XtKm15_TALE1 | HD YD NI NG NG NN YK NG HD NG NG ND NG QD NH HD | |
| XtKm12_TALE1 | HD YD NI NG NG NN YK NG HD NG NG ND NG QD NH HD | |
| XtFa1_TALE1 | HD YD NI NG NG NN YK NG HD NG NG ND NG QD NH HD | |
| Xtu 4699_TALE1 | HD YD NI NG NG NN YK NG HD NG NG ND NG QD NH HD | |
| XtLr8_TALE3 | HD YD NI NG NG NN YK NG HD NG NG ND NG QD NH HD | |
| ICMP 11055_TALE1 | HD YD NI NG NG NN YK NG HD NG NG ND NG QD NH HD | |
| P3_TALE1 | HD YD NI NG NG NN YK NG HD NG NG ND NG QD NH HD | |
| XtKm9_TALE1 | NN HD NG NG NG NN YK NG HD NG NG ND NG HD NH HD | |
| DSM 18974_TALE1 | NN HD NG NG NG NN YK NG HD NG NG ND NG HD NH HD | |
| **Class 3** | **1 2 3 4 5 6 7 8 9 10 11 12 13 14 15 16 17 18 19 20 21 22** | |
| XtKm8_TALE3 | NN HD NG NI HD HD QD | |
| XtKm34_TALE4 | NN HD NG NI HD HD HD | |
| **Class 4** | **1 2 3 4 5 6 7 8 9 10 11 12 13 14 15 16 17 18 19 20 21 22** | |
| XtKm9_TALE3 | NI NG HN NK HD NH HN HD HD HD HD QD | |
| XtKm8_TALE1b | NI NG HN NK HD NH HN HD HD HD HD QD | |
| XtKm34_TALE2 | NI NG HN NK HD NH HN HD HD HD HD QD | |
| **Class 5** | **1 2 3 4 5 6 7 8 9 10 11 12 13 14 15 16 17 18 19 20 21 22** | |
| NXtc01_TALE2 | NN NN KI NN HD NG HD NG NG NK HD HD NN QD NG QD | |
| CFBP 2541_Plasmid_TALE2 | NN NN KI NN HD NG HD NG NG NK HD HD NN QD NG QD | |
| **Class 6** | **1 2 3 4 5 6 7 8 9 10 11 12 13 14 15 16 17 18 19 20 21 22** | |
| NXtc01_TALE1 | NS KI NN HD NK GI HD NK HD NN HD NK | |
| CFBP 2541_TALE1 | NS KI NI HD NK GI HD NK HD NN HD NK | |
| **Class 7** | **1 2 3 4 5 6 7 8 9 10 11 12 13 14 15 16 17 18 19 20 21 22** | |
| XtKm15_TALE4 | NH NN HD NN HD NH HD YK NG NH Y* HD NN NI NG QD | |
| XtLr8_TALE6 | NH NN HD NN HD NH HD YK NG NH Y* HD NN NI NG QD | |
| DSM 18974_TALE3/8 | NH NN HD NN HD NH HD YK NG NH Y* HD NN NI NG QD | |
| XtKm12_TALE3 | NH NN HD NN HD NH HD YK NG NH Y* HD NN NI NG QD | |
| XtFa1_TALE5 | NH NN HD NN HD NH HD YK NG NH Y* HD NN NI NG QD | |
| ICMP 11055_TALE5 | NH NN HD NN HD NH HD YK NG NH Y* HD NN NI NG QD | |
| Xtu 4699_TALE4 | NH NN HD NN HD NH HD YK NG NH Y* HD NN NI NG QD | |
| LW16_TALE4 | NH NN HD NN HD NH HD YK NG NH Y* HD NN NI NG QD | |
| P3_TALE4 | NH NN HD NN HD NH HD YK NG NH Y* HD NN NI NG QD | |
| **Class 8** | **1 2 3 4 5 6 7 8 9 10 11 12 13 14 15 16 17 18 19 20 21 22** | |
| DSM 18974_TALE2b/3 | NN HD NG NI HN KG NI HD NI NH NG NN HD HD NI NN NI HD QD | |
| XtKm34_TALE3b | NN HD NG NI HN KG NI HD NI NH NG NN HD HD NI NN NI HD QD | |
| XtKm8_TALE4b | NN HD NG NI HN KG NI HD NI NH NG NN HD HD NI NN NI HD QD | |
| XtKm9_TALE2a | NN QD NG NN HN KG NI HD NI NH NG HN HD HD NI NN HD | |
| **Class 9** | **1 2 3 4 5 6 7 8 9 10 11 12 13 14 15 16 17 18 19 20 21 22** | |
| ICMP 11055_TALE2 | NN HD NG NN HN KG NI HD NI HN HD HN HD Y* NG HD HD HN | |
| LW16_TALE2 | NN HD NG NN HN KG NI HD NI HN HD HN HD HD NI HN HN HD | |
| XtFa1_TALE2 | NN HD NG NN HN KG NI HD NI NN HD HN HD HD NI HN HD QD | |
| P3_TALE2 | NN HD NG NN HN KG NI HD NI NN HD HN HD HD NI HN HD QD | |
| XtKm15_TALE2 | NN HD NG NN HN KG NI HD NI NN HD HN HD HD NI HN HD QD | |
| XtKm12_TALE2 | NN HD NG NN HN KG NI HD NI NN HD HN HD HD NI HN HD QD | |
| XtLr8_TALE2 | NN HD NG NN HN KG NI HD NI NN HD HN HD HD NI HN HD QD | |
| Xtu 4699_TALE2 | NN HD NG NN HN KG NI HD NI NN HD HN HD HD NI HN HD QD | |
| XtKm12_TALE5a | NN HD NG NN HN NG NI HD NI NN HD HD NN NN NI HN HD | |
| LW16_TALE6a/8 | NN HD NG NN HN NG NI HD NI NN HD HD NN NN NI HN HD | |
| XtKm8_TALE4a | NN HD NG NN HN KG NI HD NI HN HD HD NI HN NH HD HD QD | |
| XtKm34_TALE3a | NN HD NG NN HN KG NI HD NI HN HD HD NI HN NH HD HD QD | |
| **Class 10** | **1 2 3 4 5 6 7 8 9 10 11 12 13 14 15 16 17 18 19 20 21 22** | |
| XtKm8_TALE2 | NG NN HD HD NN NI HG HD ND HG NI NN HD | |
| DSM 18974_TALE4b/7 | NG NN HD HD NN NI HG HD ND HG NI NN HD | |
| XtKm9_TALE4 | NG NN HD HD NN NI HG HD ND HG NI NN HD | |
| XtKm34_TALE1 | NG NN HD HD NN NI HG HD ND HG NI NN HD | |
| **Class 11** | **1 2 3 4 5 6 7 8 9 10 11 12 13 14 15 16 17 18 19 20 21 22** | |
| P3_TALE6a /8 | NN NG HD HD HD KG NN Y* NG HD HD QD HN | |
| Xtu 4699_TALE6a/8 | NN NG HD HD HD KG NN Y* NG HD HD QD HN | |
| XtFa1_TALE3a | NN NG HD HD HD KG NN Y* NG HD HD QD HN | |
| XtLr8_TALE4a | NN NG HD HD HD KG NN Y* NG HD HD QD HN | |
| **Class 12** | **1 2 3 4 5 6 7 8 9 10 11 12 13 14 15 16 17 18 19 20 21 22** | |
| P3_TALE5a/6 | HD HN HN HD NH NH HG HD KG NN Y* NG HD HD HN | |
| LW16_TALE5a/6 | HD HN HN HD NH NH HG HD KG NN Y* NG HD HD HN | |
| Xtu 4699_TALE5a/6 | HD HN HN HD NH NH HG HD KG NN Y* NG HD HD HN | |
| ICMP11055_TALE4a | HD HN HN HD NH NH HG HD KG NN Y* NG HD HD HN | |
| XtKm12_TALE4a | HD HN HN HD NH NH HG HD KG NN Y* NG HD HD HN | |
| XtKm15_TALE5a | HD HN HN HD NH NH HG HD KG NN Y* NG HD HD HN | |
| XtLr8_TALE5a | HD HN HN HD NH NH HG HD KG NN Y* NG HD HD HN | |
| XtFa1_TALE4a | HD HN HN HD NH NH HG HD KG NN Y* NG HD NI NH NG HD HN | |
| **Class 13** | **1 2 3 4 5 6 7 8 9 10 11 12 13 14 15 16 17 18 19 20 21 22** | |
| XtKm12_TALE4b | NN HD NG NN HN HN NI NI NI NH NN HD HN NH HD HD | |
| XtKm15_TALE5b | NN HD NG NN HN HN NI NI NI NH NN HD NN NH HD HD | |
| XtFa1_TALE4b | NN HD NG NN HN HN NI NI NI NH NN HD NN NH HD HD | |
| Xtu 4699_TALE5b/5 | NN HD NG NN HN HN NI NI NI NH NN HD NN NH HD HD | |
| P3_TALE5b/5 | NN HD NG NN HN HN NI NI NI NH NN HD NN NH HD HD | |
| LW16_TALE5b/5 | NN HD NG NN HN HN NN NI NI NH NN HD NN NH HD HD | |
| **Class 14** | **1 2 3 4 5 6 7 8 9 10 11 12 13 14 15 16 17 18 19 20 21 22** | |
| XtLr8_TALE1 | NN HD NG HD HD HN NF NI NF HD HD HD HN HN HD | |
| XtKm15_TALE3 | NN HD NG HD HD HN NF NI NF HD HD HD HN HN HD | |
| Xtu 4699_TALE3 | NN HD NG HD HD HN NF NI NH HD HD HD HN HN HD | |
| LW16_TALE3 | NN HD KG HD HD HN NF NI NN HD HD HD HN HN HD | |
| P3_TALE3 | NN HD NG HD HD HN NF NI NN NN HD HD HN HN HD | |
| **Class 15** | **1 2 3 4 5 6 7 8 9 10 11 12 13 14 15 16 17 18 19 20 21 22** | |
| XtKm8_TALE4d | NN HD HD HD NI NI NI NN HD HD NN NN NI NN HD | |
| DSM 18974_TALE2d/5 | NN HD HD HD NI NI NI NN HD HD NN NN NI NN HD | |
| XtKm9_TALE2b | NN HD HD HD NI NI NI NN HD HD NN NN NI NN HD | |
| XtKm34_TALE3d | NN HD HD HD NI NI NI NN HD HD NN NN NI NN HD | |
| XtFa1_TALE3b | NN HD HD HD NI NI NI HN HD HD NN NN NI NN HD | |
| LW16_TALE6b/7 | NN HD HD HD NI NI NI HN HD HD NN NN NI NN HD | |
| P3_TALE6b/7 | HN HD HD HD NI NI NI HN HD HD NN NN NI NN HD | |
| Xtu 4699_TALE6b/7 | HN HD HD HD NI NI NI HN HD HD NN NN NI NN HD | |
| XtKm12_TALE5b | HN HD HD HD NI NI NI HN HD HD NN NN NI NN HD | |
| XtKm15_TALE6b | HN HD HD HD NI NI NI HN HD HD NN NN NI NN HD | |
| XtLr8_TALE4b | HN HD HD HD NI NI NI HN HD HD NN NN NI NN HD | |
| ICMP 11055_TALE3b | HN HD HD HD NI NI NI HN HD HD NH NN NI NN HD | |
| **Class 16** | **1 2 3 4 5 6 7 8 9 10 11 12 13 14 15 16 17 18 19 20 21 22** | |
| XtKm8_TALE1a | NH NN HE NK HD NK HD YK NG NH Y* HE NA YI NG HD NN NA NG NN NN QD | |
| DSM 18974_TALE4a/6 | NG HD HD HN NG NI HG HG HD ND NN NN NI NH QD | |
| DSM 18974_TALE2a/2 | NN NI HN HD NI NH NG HN HD HD HD NI QD | |
| ICMP 11055_TALE4b | NN HD NG HD HD HG HD KG NN Y* NG NG HD HD QD HN | |
| XtKm15_TALE6a | NN HD NG HD HD HG HD KG NN KG HD HN NN HN | |
| ICMP 11055_TALE3a | NN HD NG HD NG HD HD HG HD KG NN KG HD HN QD HN NN HD | |
| XtLr8_TALE5b | NN HD NG NN Y* NG HD HD NN NH HD HD | |
| DSM 18974_TALE2c/4 | NI NG HN NN HD NG ND NK QD NH QD |  |
